# Bifactor models of psychopathology using multi‐informant and multi‐instrument dimensional measures in the ABCD study

**DOI:** 10.1002/jcv2.12228

**Published:** 2024-02-26

**Authors:** Grace R. Jacobs, Stephanie H. Ameis, Peter Szatmari, John D. Haltigan, Aristotle N. Voineskos

**Affiliations:** ^1^ Centre for Addiction and Mental Health Toronto Ontario Canada; ^2^ Institute of Medical Science Temerty Faculty of Medicine University of Toronto Toronto Ontario Canada; ^3^ Department of Psychiatry Temerty Faculty of Medicine University of Toronto Toronto Ontario Canada; ^4^ The Hospital for Sick Children Toronto Ontario Canada

**Keywords:** attention‐deficit/hyperactivity disorder, autism spectrum disorder, bifactor, externalizing, factor analysis, general p factor, impulsivity, internalizing, neurodevelopment, psychotic‐like experiences, sensitivity to punishment, sensitivity to reward, structural equation modeling

## Abstract

**Background:**

Due to limitations of categorical definitions of mental illness, there is a need for quantitative empirical investigations of the dimensional structure of psychopathology. Using exploratory bifactor methods, this study investigated a comprehensive and representative structure of psychopathology in children to better understand how psychotic‐like experiences (PLEs), autism spectrum disorder (ASD) symptoms, impulsivity, and sensitivity to reward and punishment, may be integrated into extant general factor models of psychopathology.

**Methods:**

We used seven child‐report and three parent‐report instruments capturing diverse mental health symptoms in 11,185 children aged 9–10 from the Adolescent Brain Cognitive Development^SM^ (ABCD) Study. We built on previous modeling frameworks by conducting both split sample and full sample factor analytic approaches that harnessed recent methodological advances in bifactor exploratory structural equation modeling (B‐ESEM) to examine a wide range of psychopathology measures not previously integrated into a single analysis. Validity of psychopathology dimensions was examined by investigating associations with sex, age, cognition, imaging measures, and medical service usage.

**Results:**

All four factor analytic models showed excellent fit and similar structure within informant. PLEs loaded most highly onto a general psychopathology factor, suggesting that they may reflect non‐specific risk for mental illness. ASD symptoms loaded separately from attention/hyperactivity symptoms. Symptoms of impulsivity and sensitivity to reward and punishment loaded onto specific factors, distinct from externalizing and internalizing factors. All identified factors were associated with clinically relevant risk factors, providing preliminary evidence for their construct validity.

**Conclusion:**

By integrating diverse child‐report and parent‐report psychopathology measures for children in the ABCD sample, we deliver data on the quantitative structure of psychopathology for an exceptionally large set of measurements and discuss implications for the field.

## Graphical abstract

This study investigated a comprehensive and representative structure of psychopathology in children to better understand how psychotic‐like experiences (PLEs), autism spectrum disorder (ASD) symptoms, impulsivity, and sensitivity to reward and punishment, may be integrated into extant general factor models of psychopathology. We used seven child‐report and three parent‐report instruments capturing diverse mental health symptoms in 11,185 children aged 9–10 from the Adolescent Brain Cognitive Development^SM^ (ABCD) Study. We conducted split sample and full sample factor analytic approaches that harnessed bifactor exploratory structural equation modeling (B‐ESEM).

**Key points**

- Investigating the continuous structure of psychopathology is an important alternative to examining categorical diagnoses and critical for improving our understanding of emerging mental illnesses.
- However, studies often focus on a single psychopathology instrument, subset of measures, or specific clinical population.
- The significance of the present study is that we harnessed recent methodological advances in bifactor exploratory structural equation modeling (B‐ESEM) to examine a wide range of psychopathology measures not previously integrated into a single analysis from ten child‐report and parent‐report instruments for over 11,000 children from the population‐based Adolescent Brain Cognitive Development^SM^ (ABCD) Study.
- Findings provide insight into relationships between psychotic‐like experiences, autism spectrum disorder symptoms, impulsivity, sensitivity to reward and punishment, and a general p factor.

## INTRODUCTION

Childhood and adolescence correspond to a time of life when varying degrees of psychopathology emerge and become more prominent in children with neurodevelopmental disorders. As an alternative to categorical conceptualizations of symptoms (i.e., DSM diagnoses), factor analytic studies investigating a hierarchical and continuous structure of psychopathology data have shown evidence for a dimensional *general p* factor linked to common variance across a number of mental health symptoms and clinical disorders (Caspi et al., 2014; Kotov et al., 2017). In addition to this general higher‐order factor, specific lower‐order factors have been identified such as: *internalizing, externalizing* (Castellanos‐Ryan et al., 2016; Patalay et al., 2015); *thought* (Afzali et al., 2018; Carragher et al., 2016; Caspi et al., 2014; Cowan & Mittal, 2020; Forbes et al., 2021; Haltigan et al., 2018; Laceulle et al., 2015; Vaidyanathan et al., 2012); and an *attention‐deficit/hyperactivity disorder (ADHD)* or *neurodevelopmental* factor (Bloemen et al., 2018; Michelini et al., 2019; Moore et al., 2020; Noordhof et al., 2015). However, findings have been mixed, as studies often focus on a single instrument, subset of measures, or specific clinical population, and do not include comprehensive and detailed coverage of common psychopathology symptoms. Alternatively, investigating a broad range of symptoms in a population‐based sample can improve our global understanding of relationships and symptom organization. This is important especially when investigating children and adolescents during a period when symptoms are often comorbid, associated with impaired cognition and daily functioning, and indicative of poor, but non‐specific clinical trajectories (Caspi et al., 2020). Furthermore, examining how psychopathology factors are related to clinically‐relevant risk measures can help clarify whether associations between specific factors and risk measures are unique from a *general p* factor.

A number of symptoms have been underrepresented in factor analytic studies of psychopathology, especially in samples of children and adolescents. The relationship between psychotic‐like experiences (PLEs) and both global and specific psychopathology remains unclear, despite the prevalence of PLEs (∼20% of children) and their prediction of poor functioning, cognitive problems, and future development of psychiatric disorders (Calkins et al., 2014; Kaymaz et al., 2012; Poulton et al., 2000; Werbeloff et al., 2012). PLEs are also closely linked with other types of psychopathology such as ADHD, ASD, anxiety and depression (Addington et al., 2011; Nourredine et al., 2021; Selten et al., 2015; Velthorst et al., 2009). Previous studies have shown inconsistent findings, including that psychosis symptoms load with mania symptoms onto a *thought* factor (Cowan & Mittal, 2020; Keyes et al., 2013; Vaidyanathan et al., 2012), or that the inclusion of a *general p* factor leads to higher loading of psychosis symptoms onto this factor over a specific factor (Caspi et al., 2014; Haltigan et al., 2018; Laceulle et al., 2015). It is also unclear how autism spectrum disorder (ASD) symptoms (e.g., repetitive behaviors) fit into psychopathology structure (Stanton et al., 2021). Recent studies have found evidence for a specific *neurodevelopmental* factor, defined by a combination of symptoms related to ASD and ADHD (e.g., restlessness, inattention), separate from *externalizing* and *internalizing* factors (Michelini et al., 2019; Moore et al., 2020). This distinct *neurodevelopmental* factor is further evident in studies including more detailed ASD measures (Bloemen et al., 2018; Noordhof et al., 2015). Children and youth with ASD are at higher risk of PLEs (Kiyono et al., 2020), and subsequent psychotic disorders (Selten et al., 2015), thus supporting the importance of establishing the relationship between these constructs. Finally, while within‐instrument factor analytic studies have been conducted on detailed measures of impulsivity (Watts et al., 2020) and sensitivity to reward and punishment (Maack & Ebesutani, 2018), these measures are also typically excluded from larger comprehensive studies. Integrating the above measures with a wide range of common symptom and behavior measures in factor analytic studies is needed to investigate a complete structure of psychopathology in developing children.

Confirmatory factor analysis (CFA) and exploratory factor analysis (EFA) approaches are often applied to examine psychopathology structure, either using a hierarchical or second‐order model. However, exploratory structural equation modeling (ESEM) approaches, including those incorporating general factors (e.g., bifactor [B] – ESEM (Morin et al., 2016)) present several advantages and reflect an optimal integration of CFA and EFA (Marsh et al., 2014). Compared to a strictly confirmatory approach, ESEM better accounts for the realistic substantive and psychometric multidimensionality of measures through cross‐loadings between items, while still allowing a priori assumptions of item loading through target rotation (Morin et al., 2016). Despite these advantages, previous studies using a traditional split sample exploratory and confirmatory approach typically combine ESEM with a B‐CFA, which assumes cross‐loadings to be exactly zero, instead of using a B‐ESEM framework for confirmatory analyses. Additionally, it is unclear whether a full sample approach based on a priori knowledge using only a single B‐ESEM (which has the advantage of harnessing the entire sample size) would result in a similar factor structure as a data‐driven split sample approach.

Prior empirical work examining the structure of psychopathology in the population‐based Adolescent Brain Cognitive Development^SM^ (ABCD) cohort (Brislin et al., 2021; Clark et al., 2021; Michelini et al., 2019; Moore et al., 2020; Watts et al., 2021) has found support for a hierarchical structure with a *general p* factor. There is also evidence that identified dimensions are associated with a wide range of risk factors (Clark et al., 2021). However, these analyses have been limited to modeling psychopathology data only from a subset of available psychopathology instruments (i.e., Achenbach System of Empirically Based Assessment [ASEBA] scales). In contrast, the present study leverages the diverse mental health data available in this large sample by using 10 dimensional child‐report and parent‐report instruments. We aim to investigate a more comprehensive and representative structure of psychopathology in children that includes symptoms that are typically underrepresented in studies or have not been previously examined together. More specifically, we sought to better understand how psychotic‐like experiences (PLEs) and autism spectrum disorder (ASD) symptoms, as well as behavioral measures of impulsivity and sensitivity to reward and punishment, relate to other measures of psychopathology and a *general p* factor. We build on modeling frameworks used in prior literature, by conducting a split sample factor analytic approach that uses an exploratory ESEM and confirmatory B‐ESEM to allow realistic cross‐loadings between items, while still enforcing a structure. We additionally build on previous approaches by also conducting a full sample single B‐ESEM to determine whether findings are consistent with a split sample analysis. We hypothesize that we will be able to rigorously model the dimensional structure of childhood psychopathology by including symptoms that are often excluded. Additionally, we hypothesize that our B‐ESEM technique will be an effective approach to identify an appropriately fitting structure.

## METHODS

### Participants and ethical considerations

Psychopathology data was drawn from the ABCD Study® (data release 3.0), a large population‐based study recruited across 21 sites in the United States involving 11,875 children aged 9–10 at baseline (https://abcdstudy.org/, Barch et al., 2018). It is important to note that the range of ASD symptoms represented is restricted, as children with more severe ASD attending special‐needs schools were not included. Informed consent and assent were obtained from children and their parent(s)/legal guardian(s) in accordance with each data collection site's institutional review board. Data was accessed through the National Institute of Mental Health Data Archive. A total sample of 8839 participants for the child‐report analyses and 11,185 for the parent‐report analyses was obtained (Table 1). Children were excluded separately for child‐report and parent‐report analyses if they were missing any psychopathology measures.

**TABLE 1:** Demographics of included participants for each split and full sample analysis using child‐report and parent‐report measures.

	Child‐report samples				Parent‐report samples			
	First half	Second half	Full sample	*p* value	First half	Second half	Full sample	*p* value
*N*	4420	4419	8839		5590	5595	11,185	
Sex (F)	2105 (48%)	2100 (48%)	4205 (48%)	n.s	2664 (48%)	2670 (48%)	5334 (48%)	n.s
Age (months)	119.2 (7.5)	119.2 (7.5)	119.2 (7.5)	n.s	119.0 (7.47)	118.9 (7.59)	118.9 (7.54)	n.s
Crystalized composite t‐score	51.6 (11.3)	51.4 (11.4)	51.5 (11.3)	n.s	51.0 (11.2)	51.0 (11.2)	51.0 (11.21)	n.s
Fluid composite t‐score	46.1 (11.3)	46.4 (11.0)	46.2 (11.2)	n.s	45.7 (11.1)	46.0 (11.1)	45.8 (11.1)	n.s
Total brain volume	1,214,236 (113,503.4)	1,212,395 (111,821.5)	1,213,317 (112,663.6)	n.s	1,208,773 (112,566.7)	1,209,957 (113,181.9)	1,209,364 (112,870.6)	n.s
CBCL externalizing t‐Score	45.3 (10.2)	45.47 (10.1)	45.4 (10.1)	n.s	45.8 (10.2)	45.4 (10.3)	45.6 (10.2)	n.s
CBCL internalizing t‐Score	48.15 (10.5)	48.36 (10.4)	48.2 (10.4)	n.s	48.5 (10.6)	48.3 (10.5)	48.4 (10.5)	n.s

*Note*: Means for each of the measures are shown with standard deviations in brackets. First and second half samples for the split sample analyses were created based on matching participants on sex, age, and site. *T*‐tests and chi‐squared tests were used to compare first and second half samples on continuous and binary measures respectively.

### Psychopathology measures

All available dimensional instruments measuring baseline psychopathology in the ABCD study (Table S1) that were assessed by the year 1 timepoint were included in analyses. General and specific factors of psychopathology have been shown to be relatively stable in the same individual over time in childhood and adolescence, indicating that this relatively small amount of heterogeneity in the timing of assessments would not impact factor structure (Castellanos‐Ryan et al., 2016; Gluschkoff et al., 2019; McElroy et al., 2018; Snyder et al., 2017). This range in timepoints (baseline, 6 months, 1 year) allowed for the inclusion of a greater number of instruments and provided more comprehensive coverage of cross‐sectional measures of psychopathology.

### Child‐report items

A total of 106 child‐report items were included from seven instruments. The Prodromal Questionnaire Brief Child Version (PQ‐BC) (Loewy et al., 2011) is a 21‐item questionnaire assessing PLEs. Responses to the PQ‐BC were weighted by the amount of distress they caused children on a scale from 1 to 5. The Modified Negative Urgency, (lack of) Premeditation, (lack of) Perseverance, Sensation Seeking, Positive Urgency (UPPS‐P) for Children from PhenX is a 20‐item questionnaire assessing predisposition to impulsive actions (Lynam, 2013; Zapolski et al., 2010). The Youth Behavioral Inhibition/Behavioral Approach System Scales Modified from PhenX (BIS/BAS) is a 20‐item questionnaire assessing motivation systems related to sensitivity to reward and punishment based on Gray's Reinforcement Sensitivity Theory (Carver & White, 1994; Cooper et al., 2007). The Delinquency Scale is a 10‐item shortened assessment from a scale developed for use in the Causes and Correlated of Delinquency Program (Hoeve et al., 2008; Theobald et al., 2014). The National Institutes of Health (NIH) Toolbox Positive Affective Items is a 9‐item questionnaire assessing positive feeling states, meaning, and life satisfaction (Gershon et al., 2013; Salsman et al., 2013). The Brief Problem Monitor (BPM) is an 19‐item assessment of internalizing, externalizing and attention problems taken from the Child Behavior Checklist (CBCL) (Achenbach, 2009). The Mania 7‐Up scale is a 7‐item questionnaire assessing manic symptoms taken from the General Behavior Inventory (Youngstrom et al., 2013) (see Table S1 for details).

### Parent‐report items

A total of 140 parent‐report items were included from three instruments. The CBCL is a 119‐item questionnaire (ages 6–18) assessing a range of behavioral and emotional problems (Achenbach, 2009). The shortened version of the Social Responsiveness Scale (SRS) is a 11‐item questionnaire assessing ASD symptoms such as social deficits, communication deficits and repetitive behaviors (Reiersen et al., 2008). The Mania Scale is a 10‐item questionnaire including items related to hypomania and biphasic symptoms from the Parent General Behavior Inventory (GBI) (Youngstrom et al., 2001).

### Validation measures

Associations between each psychopathology dimension from all models and validation measures related to clinical risk were evaluated. Validation measures included global imaging measures of cortical thickness, subcortical volume, and cortical surface area; cognitive measures of fluid and crystalized cognitive performance; and medical service usage (parent‐report). Fluid and crystalized t‐score composites were based on performance on the NIH Toolbox Cognitive Battery. ABCD imaging procedures and quality control have been previously described in detail (Hagler et al., 2019). All participants included in this study were scanned on a Siemens, Phillips or GE Healthcare 3T scanner (Prisma, Prisma Fit, or Discovery MR750) using a 32‐channel head coil. T1‐weighted MRI scans were processed using Freesurfer v5.3. If more than one scan was collected, only one was used for processing following manual quality control. Imaging data from participants were not used if they failed ABCD's manual inspection of the structural scan or Freesurfer's cortical surface reconstruction.

### Statistical analyses

We used two factor analytic methods that were applied separately for child–report and parent‐report instruments. First, we conducted a split sample approach. Our approach used an ESEM in the first half of the sample and a B‐ESEM in the second half of the sample instead of a more traditional CFA. A CFA restricts an item's variance to a single factor and limits item loadings to be exactly zero for all factors that items are not previously found to load highly on. This is a strongly restrictive assumption, particularly for multidimensional measures used in psychiatry (Morin et al., 2016). By alternatively using a B‐ESEM in the second half of the sample, we allow realistic cross‐loadings between items, while still enforcing a structure based on the ESEM in the first half of the sample. Secondly, we conducted a full sample approach consisting of only a single B‐ESEM, with the option to make minimal revisions to the model. We took this opportunity to flexibly test an a priori structure using a B‐ESEM, while leveraging the entire sample. By applying both split and full sample approaches, we can compare findings between a data‐driven factor structure and an a priori factor structure.

All factor analyses were conducted in Mplus Version 7.11 (Muthen & Muthen, 2012). The mean‐ and variance‐adjusted weighted least squares (WLSMV) estimator was used, as data was ordered‐categorical (Li, 2016). Models were identified by fixing the variance of each factor to 1, and freely estimating the first factor loading. Specific factors were orthogonal to each other and the general factor (Chen et al., 2012; Reise, 2012). Consistent with prior work (Michelini et al., 2019; Moore et al., 2020), psychopathology symptom items were removed from analyses if they had a low endorsement (>99.5% of children didn't endorse them). The CBCL item “wishes to be of opposite sex” was additionally removed because it is no longer considered to reflect psychopathology. If separate items had a polychoric correlation of >0.85, they were aggregated into a single item by taking the rounded average value across items for each participant (Michelini et al., 2019; Moore et al., 2020). See Supporting Information S1: Methods for item exclusion details. Clustering within family, stratification based on data collection site, and confirmatory factors accounting for shared variance based on instrument (method factors) were included in all models (Moore et al., 2020). Open‐source code is available for analyses at https://github.com/gracejacobs/ABCD_factor_analysis.

Model fit was analyzed using the magnitude of factor loadings, root‐mean‐square error of approximation (RMSEA), Tucker‐Lewis index (TLI), and the comparative fit index (CFI). RMSEA values less than 0.05 indicate an ideal close model fit, while TLI and CFI values above 0.95 indicate a very good fit (Brown, 2015). Items were considered to load onto a factor when their standardized loadings were 0.32 or greater (Tabachnick & Fidell, 2001). This cut off was chosen to indicate sufficient unique variance given the partial variance accounted for by the method factors. Demographics for participants included in each of the analyses are shown in Table 1.

### Split sample analyses

The first analytic approach conducted ESEM in the first half of the sample to determine the optimal number of factors and item loadings, similar to the split sample modeling approach taken in Moore et al., 2020. The total sample was randomly split by site, sex, and age into exploratory and confirmatory subsamples for both child‐report and parent‐report models, following the removal of children with missing data. For the ESEM analyses in the first half of the sample we did not specify any a priori target loadings, but rather explored loadings for each item across a range of factor numbers using an OBLIMIN rotation (Moore et al., 2020). Parallel analysis (Horn, 1965) with Glorfeld correction (Glorfeld, 1995) indicated that up to 14 factors for child‐report analyses and 19 factors for parent‐report analyses could be extracted. The minimum average partial criterion (Velicer, 1976) indicated that up to 11 factors for child‐report analyses and 13 factors for parent‐report analyses could be extracted. We extracted 4 interpretable factors for child‐report analyses (in addition to 7 methods factors) and 5 interpretable factors for parent‐report analyses (in addition to 3 methods factors), in which the majority of items had a loading of >0.32 on at least one factor. This aligns with previous factor analytic studies examining psychopathology structure using similar measures that found up to five specific factors (Carragher et al., 2016; Caspi et al., 2014; Haltigan et al., 2018; Laceulle et al., 2015; Michelini et al., 2019; Moore et al., 2020; Noordhof et al., 2015). The identified factor structure was confirmed using B‐ESEM with target rotations in the second half of the sample, which allowed item loadings to be targeted onto specific factor dimensions and cross‐loadings to be targeted to as close as possible to zero (but no forced zero loadings). We target loaded items onto specific factors for the B‐ESEM when their standardized loadings were greater than 0.32 in the exploratory analysis (Tabachnick & Fidell, 2001).

### Full sample analyses

**FIGURE 1:**
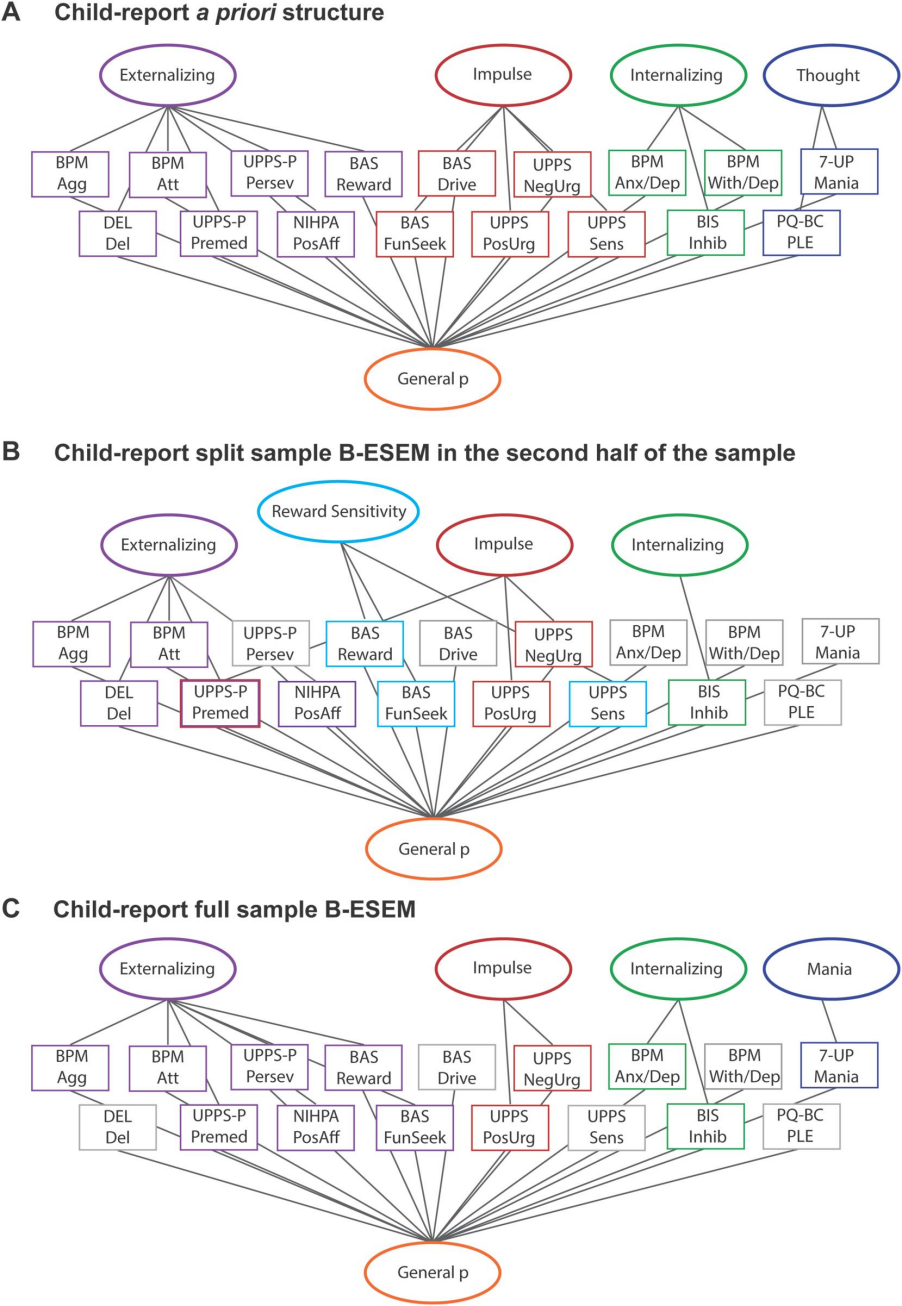
A priori factor structure for child‐report psychopathology items, followed by split and full sample analysis factor structure findings. Black lines indicate if any items had loadings of 0.32 or greater onto a factor. Agg, Aggressive Behavior; Anx/Dep, Anxious/depressed; Att, Attention Problems; Del, Delinquency; Inhib, Inhibition/punishment sensitivity; NegUrg, Negative Urgency; Persev, Lack of Perseverance; PLEs, Psychotic‐like experiences; PosAff, Positive Affective; PosUrg, Positive Urgency; Premed, Lack of Premeditation; Reward, Reward Responsiveness; Sens, Sensation seeking; With/dep, Withdrawn/depressed.

**FIGURE 2:**
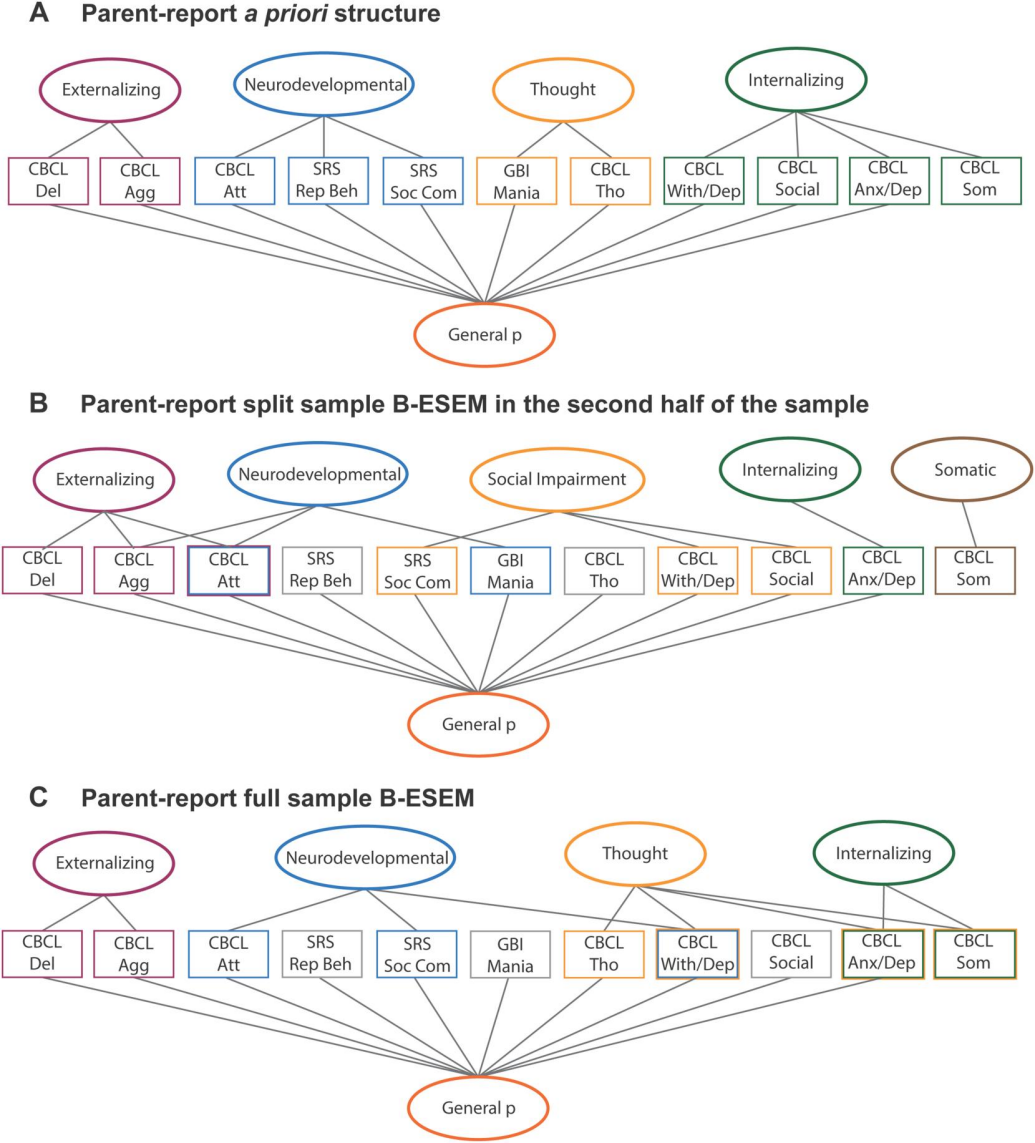
A priori factor structure for parent‐report psychopathology items, followed by split and full sample analysis factor structure findings. Black lines indicate if any items had loadings of 0.32 or greater onto a factor. Agg, Aggressive Behavior; Anx/Dep, Anxious/depressed; Att/NDD=Attention Problems & Neurodevelopmental; Del, Rule‐breaking behavior; Rep Beh, Repetitive behaviors; Soc Com, Social Communication Impairment; Tho, Thought; With/dep, Withdrawn/depressed.

The second analytic approach taken to identify psychopathology factor structure leveraged the entire ABCD sample by conducting B‐ESEM in the full sample separately for child‐report and parent–report items, using a priori conceptually‐based target rotation (see Figures 1A and 2A, Supporting Information S1: Methods for details) (Morin et al., 2016). Following examination of this initial B‐ESEM model for each informant, a small number of item target loadings were reassessed and adjusted to optimize the final model if needed. Indicators of non‐target factors were specified to be as close to zero (but non‐zero) as possible, which allowed us to hypothesize the number of factors and target loadings of items onto each factor, while letting all items possess non‐zero cross‐loadings onto nonfocal factors (Marsh et al., 2014).

### Validation measure analyses

Patterns of associations between psychopathology dimensions and validation measures related to clinical risk were examined using simultaneous regressions of validation measures onto the general and specific factors in latent space within each structural equation model in Mplus. Associations between risk measures and dimensions in all four child‐report and parent‐report models were compared to: (1) generally understand how patterns of covariance across psychiatric symptoms in children captured by these factors related to risk measures; (2) determine if previous findings using a subset of measures and alternative modeling approaches (Brislin et al., 2021; Durham et al., 2021; Michelini et al., 2019; Moore et al., 2020) were replicated; and (3) provide insight into whether one or more of the models examined in this study was more relevant to validation risk measures. Associations were examined in the full sample for full sample analyses and in the second half of the sample for split sample analyses. Sex and age were included as covariates. To additionally compare models, pearson correlations were examined between *general p, externalizing* and *internalizing* factor scores computed using the maximum a posteriori method and saved from each of the four factor structure models.

## RESULTS

### Child‐report split sample analyses

A five‐factor (RMSEA = 0.018, CFI = 0.955, TLI = 0.948) model found using ESEM showed the best fit in the first half of the sample for child‐report items as compared to models with fewer factors (see Supporting Information S1: Results for specific factor details). The four‐factor (RMSEA = 0.018, CFI = 0.956, TLI = 0.950) and five‐factor (RMSEA = 0.016, CFI = 0.964, TLI = 0.959) models found using B‐ESEM in the second half of the sample (with item loadings targeted based on ESEM findings in the first half of the sample and the addition of a *general p* factor) showed excellent fits. However, only a single item (I enjoy taking risks) loaded onto the *mania* factor in the five‐factor solution. As only four interpretable specific factors were extracted with more than 1 item with a loading ≥0.32 on each factor, a four‐factor model excluding the *mania* factor was used for target rotation in the final bifactor model.

In the final four‐factor (plus a *general p*) model (Figure 1B, Table S2), the *externalizing* factor was defined by 14 attention problems and aggressive behavior items (e.g., trouble concentrating, argues a lot), 2 delinquency items (carries hidden weapon, steals, hits people), 2 lack of premeditation items (I like/tend to stop and think before doing things), and negative loadings on 4 positive affective items (calm, confident, concentrated, at ease). The *internalizing* factor was defined by 5 sensitivity to punishment items (e.g., I feel worried when I do poorly). Related anxious/depressed items loaded just under the 0.32 threshold (e.g., I worry a lot, I am self‐conscious). The *reward sensitivity* factor was defined by 5 reward responsiveness items (e.g., thrilled when good things happen), 3 fun‐seeking items (e.g., tend to act in the spur of the moment) and 2 sensation seeking items (e.g., I like new thrilling things). The *impulse* factor was defined by 4 positive urgency (e.g., I act without thinking when I am very happy), 4 negative urgency items (e.g., when I am upset I act without thinking) and 1 lack of premeditation item (I tend to stop and think before doing things). The *general p* factor was most clearly indexed by 53 items with the highest loadings of items related to mania, aggressive behavior, delinquency, anxious/depressed, and attention problems. Most PLEs loaded ≥0.32 onto the *general p* factor, as well as impulsivity items related to urgency. Average factor loadings for the four‐factor model were 0.44 for the *externalizing* factor, 0.47 for the *internalizing* factor, 0.43 for the *reward sensitivity* factor, 0.51 for the *impulse* factor, and 0.43 for the *general p* factor.

### Child‐report full sample analyses with a priori conceptual target loadings

The conceptually‐based B‐ESEM in the full sample using child‐report items showed a good (TLI = 0.947) to very good fit (RMSEA = 0.019, CFI = 0.953) (Figure 1C, Table S3) after minor adjustments to 5 items were made to the original a priori model (see Supporting Information S1: Results). Note that goodness of fit did not change between models as rotation does not impact these indices.

The *externalizing* factor was defined by 10 attention and aggressive items (e.g., difficulties concentrating, inattention, argues a lot) and 8 impulsivity items related to lack of premeditation (e.g., I like to stop to think before acting) and lack of perseverance (e.g., I tend to get the job done on time). Items related to positive affect (confident, concentrate), as well as 5 reward sensitivity (e.g., I get thrilled when good things happen) and fun seeking items (e.g., I crave excitement and new sensations) negatively loaded onto the *externalizing* factor. The *internalizing* factor was defined by 5 inhibition items (e.g., I worry about making mistakes) and 2 anxious/depressed items (self‐conscious, worry a lot). The *impulse* factor was defined by 8 impulsivity items related to positive urgency (e.g., I tend to lose control when I am in a great mood) and negative urgency (e.g., when I feel rejected I often say things that I later regret). The *mania* factor was defined by 6 mania items (e.g., periods of extreme happiness and high energy lasting several days or more when they felt they were a very important person or that their abilities or talents were better than most other people's). The *general p* factor was most clearly indexed by 55 items. Attention problems, aggressive behavior, anxious/depressed, delinquency, and mania items loaded most highly on the *general p* factor, followed by fun seeking, PLEs, and reward sensitivity items. Very few impulsivity and inhibition items, and no positive affective items loaded onto the *general p* factor. The average loadings were 0.39 for the *externalizing* factor, 0.40 for the *internalizing* factor, 0.53 for the *impulse* factor, 0.38 for the *mania* factor, and 0.44 for the *general p* factor.

### Parent‐report split sample analyses

The five‐factor (RMSEA = 0.015, CFI = 0.963, TLI = 0.960) model found using ESEM in the first half of the sample showed the best fit compared to models with fewer factors (see Supporting Information S1: Results for specific factor details). The five‐factor model found using B‐ESEM in the second half of the sample (with item loadings targeted based on ESEM findings in the first half of the sample and the addition of a *general p* factor) showed an excellent fit (RMSEA = 0.014, CFI = 0.970, TLI = 0.966). The *externalizing* factor was defined by 23 aggressive behavior and delinquent items (e.g., cruel, disobedient), 1 attention item (impulsive) and 1 social item (doesn't get along with others). The *internalizing* factor was defined by 8 anxious/depressed items (e.g., has to be perfect, too fearful or anxious, feels worthless). The *neurodevelopmental* factor was defined by 4 attention items (restless, impulsive, distracted), 2 aggressive items (shows off, talks too much), 1 mania item (racing thoughts). The *social impairment* factor was defined by 7 social items (e.g., doesn't get along well with others, resistant to change, narrow range of interests) and 2 withdrawn/depressed items (rather be alone, withdrawn). The *somatic* factor was defined by 6 somatic and other problems (e.g., nausea, trouble sleeping). Nearly all items loaded onto the *general p* factor. The average factor loadings were 0.47 for the *externalizing* factor, 0.4 for the *neurodevelopmental* factor, 0.42 for the *internalizing* factor, 0.44 for the *social impairment* factor, 0.4 for the *somatic* factor, and 0.57 for the *general p* factor (Figure 2B, Table S4).

### Parent‐report full sample analyses with a priori conceptual target loadings

The conceptually‐based B‐ESEM in the full sample using parent‐report items showed an excellent fit (RMSEA = 0.016, CFI = 0.961, TLI = 0.957) (Figure 2C, Table S5). The *externalizing* factor was defined by 25 aggressive behavior and delinquent items (e.g., disobedience, threatens people, cruel, steals). The *internalizing* factor was defined by 10 anxious/depressed items (e.g., worries, has to be perfect) and 3 somatic items (feels dizzy, nausea, stomach‐aches). The *thought* factor was defined by 2 thought items (sees things that aren't there, hears sounds or voices) and negative loadings of 3 anxious/depressed, social, and somatic items (not liked, feels too guilty, overtired without reason). The *neurodevelopmental* factor was defined by 4 attention problems and neurodevelopmental items (restless, talks too much, easily distracted, shows off) and negative loadings of 1 social impairment item (rather be alone) and 3 withdraw/depressed items (too shy, withdrawn, rather be alone). The items with the highest loadings on the *general p* factor were CBCL items (e.g., strange behavior, not liked, impulsive, mood changes, obsessions, repetitive acts). Most items loaded onto the *general p* factor. The highest loading items were related to thought, aggressive behavior, social problems, and attention problems. The average loadings were 0.44 for the *externalizing* factor, 0.42 for the *internalizing* factor, 0.40 for the *thought* factor, 0.39 for the *neurodevelopmental* factor, and 0.58 for the *general p* factor. For all model fit indices, see Table S6.

### Validation measures

Simultaneous regressions within B‐ESEMs found that latent general and specific factors from all four child‐report and parent‐report models explained significant unique variance in the risk measures (Tables 2 and 3). Notably, the child‐report split sample model had the fewest significant associations compared to the other three models. Across all models *externalizing*, and *general p* factor scores were significantly higher in males, while *internalizing* scores were significantly higher in females. The *general p* factor scores were associated with lower fluid and crystalized cognition across all four models, and were higher for individuals with greater medical service usage in the parent‐report models, similar to findings in (Michelini et al., 2019). *General p* scores were also associated with lower cortical volume across models similar to findings in (Durham et al., 2021). See Supporting Information S1: Results for a detailed description of findings for all factors, as well as results for correlations between derived factors (computed as factor scores) across the models.

**TABLE 2:** Child‐report full and split sample models and validation measures.

	Full sample child‐report B‐ESEM									
	EXT		INT		MAN		IMP		General P	
	B	*p*	B	*p*	B	*p*	B	*p*	B	*p*
Age (months)	0.033	0.07	−0.002	0.907	−0.085	**<0.001**	−0.052	**0.002**	−0.017	0.335
Sex	0.184	**<0.001**	−0.379	**<0.001**	0.162	**<0.001**	0.207	**<0.001**	0.34	**<0.001**
Crystalized cognition	0.208	0.097	−0.023	0.877	−1.591	**<0.001**	−0.703	**<0.001**	−0.413	**0.001**
Fluid cognition	−0.413	**0.001**	−0.557	**<0.001**	−0.376	**0.02**	−0.143	0.246	−0.407	**0.001**
Subcortical volume	−0.084	0.056	−0.102	**0.02**	0.072	0.186	0.029	0.472	0.007	0.873
Surface area	−0.429	**0.008**	0.156	0.379	0.594	**0.004**	−0.32	**0.041**	0.34	**0.033**
Cortical volume	0.148	**0.011**	−0.034	0.585	−0.443	**<0.001**	0.04	0.473	−0.32	**<0.001**
Medical service use	0.065	**<0.001**	−0.011	0.544	−0.044	**0.047**	−0.04	**0.012**	0.002	0.906

	Split Sample Child‐Report B‐ESEM in Second Half of Sample									
	EXT		INT		IMP		REW		General P	
	B	*p*	B	*p*	B	*p*	B	*p*	B	*p*
Age (months)	0.049	0.09	0.009	0.724	−0.039	0.117	0.003	0.915	−0.063	**0.025**
Sex	0.16	**0.002**	−0.359	**<0.001**	0.205	**<0.001**	0.049	0.328	0.382	**<0.001**
Crystalized cognition	0.693	**0.001**	−0.018	0.929	−0.518	**0.004**	0.896	**<0.001**	−1.174	**<0.001**
Fluid cognition	−0.167	0.408	−0.474	**0.015**	−0.207	0.249	0.587	**0.003**	−0.678	**<0.001**
Subcortical volume	−0.183	**0.006**	−0.12	0.052	−0.059	0.309	0.051	0.422	0.018	0.788
Surface area	−0.573	**0.026**	0.184	0.473	−0.407	0.07	−0.121	0.631	0.267	0.292
Cortical volume	0.3	**0.001**	−0.008	0.933	0.103	0.213	0.105	0.252	−0.38	**<0.001**
Medical service use	0.077	**0.004**	0.03	0.227	0.015	0.514	−0.005	0.849	−0.012	0.63

*Note*: Standardized beta weights (B) and *p* values (*p*) from regression analyses with each factor. The bolded values indicated significant associations (*p* < 0.05).

Abbreviations: EXT, externalizing; INT, internalizing; MAN, mania; IMP, impulse; REW, reward sensitivity.

**TABLE 3:** Parent‐report full and split sample models and validation measures.

	Full sample parent‐report B‐ESEM									
	EXT		INT		THO		NDD		General P	
	B	*p*	B	*p*	B	*p*	B	*p*	B	*p*
Age (months)	−0.058	**0.003**	−0.009	0.633	0.003	0.875	−0.087	**<0.001**	0.003	0.875
Sex	0.202	**<0.001**	−0.556	**<0.001**	0.383	**<0.001**	0.146	**0.017**	0.383	**<0.001**
Crystalized cognition	−0.682	**<0.001**	0.664	**<0.001**	−0.28	**0.022**	−0.734	**<0.001**	−0.28	**0.022**
Fluid cognition	0.545	**<0.001**	0.823	**<0.001**	−1.256	**<0.001**	−0.248	0.187	−1.256	**<0.001**
Subcortical volume	−0.072	0.125	−0.06	0.186	−0.048	0.218	0.008	0.851	−0.048	0.218
Surface area	−0.807	**<0.001**	−0.258	0.15	0.046	0.755	−0.114	0.518	0.046	0.755
Cortical volume	0.163	**0.007**	0.258	**<0.001**	−0.202	**<0.001**	−0.032	0.639	−0.202	**<0.001**
Medical service use	0.003	0.869	0.172	**<0.001**	0.084	**0.001**	0.14	**<0.001**	0.207	**<0.001**

	Split Sample Parent‐Report B‐ESEM in Second half of the Sample											
	EXT		INT		NDD		SOC		SOM		General P	
	B	*p*	B	*p*	B	*p*	B	*p*	B	*p*	B	*p*
Age (months)	−0.025	0.367	−0.006	0.846	−0.025	0.347	0.107	**<0.001**	0.03	0.322	0	0.993
Sex	0.402	**<0.001**	−0.45	**<0.001**	0.447	**<0.001**	0.149	**0.007**	−0.111	0.07	0.182	**<0.001**
Crystalized cognition	−0.63	**0.002**	1.085	**<0.001**	−0.326	0.112	0.661	**0.002**	−0.61	**0.008**	−0.051	**0.004**
Fluid cognition	0.181	0.374	0.856	**<0.001**	−0.767	**<0.001**	−0.843	**<0.001**	0.57	**0.011**	−0.1	**<0.001**
Subcortical volume	−0.192	**0.003**	−0.147	**0.023**	−0.062	0.316	0.047	0.477	−0.051	0.46	−0.041	0.116
Surface area	−1.534	**<0.001**	−0.461	0.077	0.017	0.945	0.086	0.752	−0.086	0.759	0.014	0.689
Cortical volume	0.463	**<0.001**	0.601	**<0.001**	0.014	0.879	−0.051	0.601	−0.069	0.516	−0.134	**0.001**
Medical service use	−0.032	0.264	0.12	**<0.001**	0.112	**<0.001**	−0.084	**0.003**	0.148	**<0.001**	0.187	**<0.001**

*Note*: Standardized beta weights (B) and *p* values (*p*) from regression analyses with each factor. The bolded values indicated significant associations (*p* < 0.05).

Abbreviations: EXT, externalizing; INT, internalizing; THO, thought; NDD, neurodevelopmental; SOC, social impairment; SOM, somatic.

## DISCUSSION

In this investigation, we used factor analytic approaches harnessing B‐ESEM to study comprehensive psychopathology structure in the ABCD sample across multiple instruments and psychopathology measures from both child and parent informants. Goodness of fit indices indicated that the optimal factor structure in the child‐report data included *general p, externalizing, internalizing, impulse* and *reward sensitivity* factors, while the optimal factor structure in the parent‐report data included *general p, externalizing, internalizing, neurodevelopmental*, *social impairment and somatic* factors. PLEs in the child‐report models loaded most onto the *general p* factor. ASD and ADHD measures loaded together onto a *neurodevelopmental* factor in the full sample parent‐report analysis, and separately onto neurodevelopmental and social impairment factors in the split sample parent‐report analysis. Measures of impulsivity and sensitivity to reward and punishment loaded partially with items from other scales and partially on independent factors. Finally, across both child‐report and parent‐report analyses, we were able to replicate and expand upon a psychopathology structure including a *general p, internalizing, externalizing*, and additional specific psychopathology factors, that were associated with cognitive, imaging, and clinical risk measures.

### Psychotic‐like experiences (PLEs)

While recent studies have used the more limited set of ASEBA Instruments (e.g., the CBCL) to assess child psychopathology (Brislin et al., 2021; Clark et al., 2021; Michelini et al., 2019; Moore et al., 2020; Watts et al., 2021), we used the full scope of measures available in the ABCD Study, which allowed us to better investigate relationships between PLEs, ASD symptoms, impulsivity, and sensitivity to reward and punishment for the first time in a factor analytic investigation with other measures. PLEs included in the child‐report models did not load highly onto a specific *thought* factor with mania items or another factor, but largely loaded directly onto the *general p* factor consistent with prior work (Bloemen et al., 2018; Haltigan et al., 2018; Laceulle et al., 2015). This may indicate that the presence of PLEs at this stage of life is related to elevated severity for a range of types of psychopathology and may represent the ‘end‐point’ of a clinical severity spectrum, which in DSM disorders is captured by poorer functioning and outcomes. Alternatively, variance in PLEs may not have been captured by a specific factor because their presence at 9–10 years of age may be related to an increased risk for general psychopathology (both current and later expression) instead of specifically psychosis. Studies in children and adolescents have found that psychosis‐related items fit better with a *general p* factor instead of a specific *thought* factor (Bloemen et al., 2018; Haltigan et al., 2018; Laceulle et al., 2015). Whereas, multiple factor analytic investigations in adult samples have demonstrated a *thought* factor distinct from the *general p* factor (Kotov, Chang, et al., 2011; Kotov et al., 2020; Kotov, Ruggero, et al., 2011), although others have not (Caspi et al., 2014; Rosenström et al., 2019). Taken together, current findings could indicate that an underlying specific dimension of psychosis symptoms may emerge later in development.

### ASD symptoms

The tenability of a *neurodevelopmental* factor was investigated in the parent‐report models, based on the availability of two parent‐report instruments in the ABCD Study (i.e., SRS and CBCL). However, ASD symptoms from the SRS did not load strongly onto either a separate *neurodevelopmental* factor as expected, or onto *internalizing* or *externalizing* factors. Items from the SRS have been previously found to have a two‐factor structure: (1) social communication impairment and (2) repetitive, restricted behavior (Frazier et al., 2014). The *neurodevelopmental* factor that emerged in the parent‐report full sample analysis was predominantly driven by attention, hyperactivity, and social communication impairment measures. In split sample analyses, attention and hyperactivity items loaded separately from social communication impairment measures. These results are consistent with prior evidence that ASD and ADHD measures can load both together (Michelini et al., 2019) and separately, likely depending on the range of ASD measures examined, age of participants, and type of sample (i.e., clinical or population) (Bloemen et al., 2018; Michelini et al., 2019; Moore et al., 2020; Noordhof et al., 2015). While ADHD symptoms are consistently included in an externalizing spectra or super spectrum in the evolving Hierarchical Taxonomy of Psychopathology (HiTOP) framework (Kotov et al., 2017; Krueger et al., 2021), more work is needed to determine how to classify ASD symptoms. Our findings build on prior work by including a wide range of symptoms (Stanton et al., 2021) and add to increasing evidence that overlap between ASD and ADHD should be taken into account for classification.

### Impulsivity and sensitivity to reward and punishment

Impulsivity items from the UPPS‐P and sensitivity to reward and punishment items from the BIS/BAS have been associated with behavior and mental illness, but to our knowledge, this is the first time that items from these instruments have been dimensionally modeled with other measures of psychopathology. Impulsivity is highly heterogeneous and has been associated with externalizing, internalizing, ADHD and personality disorders (Berg et al., 2015). Our findings provide initial empirical support for a specific positive and negative *urgency* factor, which could fit into the proposed disinhibition spectra in the HiTOP framework (Krueger et al., 2021). A recent analysis examining these two instruments in the ABCD sample showed moderate to large correlations between urgency and sensation seeking impulsivity scores with reward sensitivity scores (Watts et al., 2020). However, our findings showed that reward sensitivity items loaded most strongly onto either the *externalizing* factor or a specific *reward sensitivity* factor, supporting other prior evidence that higher reward sensitivity scores have been linked with externalizing symptoms in adults (Hundt et al., 2008) and symptoms of ADHD in children (Becker et al., 2013). While elevated sensitivity to reward may fit with externalizing symptoms, reduced sensitivity to reward or lower BAS scores have been linked to depression and anhedonia (Allen et al., 2019; Bijttebier et al., 2009). Taken with our findings of a separate reward sensitivity factor, ultimately, reward sensitivity may fit best between an overarching internalizing and externalizing spectra within the HiTOP framework.

Lack of premeditation and perseverance impulsivity items, which may be related to lower executive or self‐control, and a high tolerance for negative consequences (Berg et al., 2015; Segarra et al., 2014), also loaded onto the *externalizing* factor, as expected (Watts et al., 2020). Although there is consistent evidence that negative urgency impulsivity and sensitivity to punishment scores are correlated (Berg et al., 2015; Watts et al., 2020), sensitivity to punishment items are strongly associated with anxiety and depression (Katz et al., 2020). Our results suggest that when measures of internalizing symptoms, such as from the BPM, are included in analyses, sensitivity to punishment items load with internalizing symptoms instead of with negative urgency impulsivity items. These series of findings provide novel insight into shared covariance across impulsivity items from the UPPS‐P and sensitivity to reward and punishment items from the BIS/BAS, as well as how they fit into the structure of psychopathology commonly studied.

### Risk factor validation via cognitive and brain structure measures

When assessed using simultaneous regressions within the SEMs, psychopathology factors across models explained unique variance for most validating risk factors and were largely consistent with previous findings. *General p* factors were most consistently significantly associated with all measures across models, aligning with prior findings (Brislin et al., 2021; Durham et al., 2021; Michelini et al., 2019; Moore et al., 2020). Findings of elevated cognition associated with the *internalizing* factor, and reduced cognition associated with both the *general p* and *neurodevelopmental* factors also converge with prior findings (Brislin et al., 2021; Michelini et al., 2019; Moore et al., 2020). Compared to clinical and cognitive measures, fewer studies have examined neural correlates of psychopathology dimensions (Durham et al., 2021; Kaczkurkin et al., 2019; Karcher et al., 2021). However, results of our analyses indicated that many factors had significant differences in global measures of cortical volume or surface area. This emphasizes the importance of further investigation into the relationships between dimensions of psychopathology and brain metrics, which can aid in understanding common neural mechanisms across disorders, as well as shared and unique factors associated with them. Further, case‐control approaches to investigate biomarkers may be confounded by variance related to overall psychopathology, and studying neural correlates of dimensions of psychopathology distinct from a *general p* factor may aid in elucidating meaningful neurobiological relationships (Parkes et al., 2021).

### Limitations

Examining psychopathology in a population‐based sample provides the opportunity to capture structure that is more generalizable than studying a clinical subsample. However, an important consideration when interpreting the structure identified in this study is that a large number of children in the ABCD Study have limited endorsement of symptoms of psychopathology and not all assessments have been designed for a population‐based sample. Our use of B‐ESEM for analyses was an optimal combination of techniques that allowed us to refine an a priori model usually tested using CFA approaches (which most often severely or completely constrain plausible item‐factor cross‐loadings). B‐ESEM more precisely accounts for the multidimensional covariance present in the sample (i.e., that variance in items might be captured by multiple dimensions of psychopathology (Morin et al., 2016)). Nonetheless, our theoretical target rotation approach is one of several rotation approaches in B‐ESEM and other approaches, such as those that implement empirical iterative rotation algorithms (e.g., see Garcia‐Garzon et al., 2019) may offer more optimal recovery of the true population factor structure present in the sample data. Additionally, our validation analyses minimally assume—rather than formally test— measurement invariance. Future conceptual and methodological work should aim to formally investigate these assumptions in the context of measurement invariance (Meredith, 1993).

## CONCLUSION

The current findings build on existing work examining the structure of psychopathology including a higher‐order *general p* factor along with specific lower‐order dimensions. Previous findings have been mixed as they often focus on a single instrument, subset of measures, or specific clinical population. We aimed to address these challenges by integrating diverse and representative child‐report and parent‐report measures for children from a large population‐based study. This allowed us to improve our global understanding of relationships between symptoms and their hierarchical organization at this early age. Examination of cognition, brain measures, and medical service usage found preliminary evidence for factor construct validity. Furthermore, our study is strengthened by the use of B‐ESEM as a confirmatory approach to identify factor structure, which presents several advantages over more traditional modeling approaches and should be continued to be harnessed in future studies examining psychopathology in children.

## AUTHOR CONTRIBUTIONS

**Grace R. Jacobs**: Conceptualization; formal analysis; investigation; methodology; project administration; visualization; writing – original draft; writing – review & editing. **Stephanie H. Ameis**: Supervision; writing – review & editing. Peter Szatmari: Writing – review & editing. John D. Haltigan: Conceptualization; formal analysis; methodology; supervision; writing – review & editing. **Aristotle N. Voineskos**: Conceptualization; data curation; methodology; resources; software; supervision; writing – review & editing.

## CONFLICT OF INTEREST STATEMENT

Peter Szatmari has received royalties from Guilford Press and Simon & Schuster. The remaining authors have declared that they have no competing or potential conflicts of interest.

## ETHICAL CONSIDERATIONS

Informed consent and assent were obtained from children and their parent(s)/legal guardian(s) in accordance with each data collection site's institutional review board. Data was accessed through the National Institute of Mental Health Data Archive.

## Supporting information

Supporting Information S1

## Data Availability

Data used in the preparation of this article were obtained from the Adolescent Brain Cognitive Development (ABCD) Study (https://abcdstudy.org), held in the NIMH Data Archive (NDA).
