# Supplementary material for: Bifactor models of psychopathology using multi‐informant and multi‐instrument dimensional measures in the ABCD study
Source: JCPP Adv. 2024 Feb 26;4(2):e12228. doi: 10.1002/jcv2.12228 (PMC11143956; doi:10.1002/jcv2.12228)
Supplement: Supplementary file 1 — Supporting Information S1 [file JCV2-4-e12228-s001.docx]

# Supporting Information

## Methods

### Excluded items for child-report analyses

#### Full sample child-report analyses (n=8839)

A total of 103 items were included in analyses from seven child-report instruments. Two items from the Delinquency Scale (#8 Have you stolen or tried to steal things worth between $5 and $50 in the past year? & #10 Have you been arrested or picked up by the police in the past year for other than a minor traffic offense?) were excluded due to low endorsement. Two items from this scale were combined due to polychoric correlations >0.85 (#5 Have you taken something from a store without paying for it in the past year? & #6 Have you stolen or tried to steal things worth $5 or less in the past year?).

#### Split sample child-report analyses (first half=4420, second half=4419)

A total of 102 items were included in analyses from seven instruments. Two items from the Delinquency Scale (#8 Have you stolen or tried to steal things worth between $5 and $50 in the past year? & #10 Have you been arrested or picked up by the police in the past year for other than a minor traffic offense?) were excluded due to low endorsement and one item was removed due to negative residual variance (#4 gang fights). Two items from this scale were combined due to polychoric correlations >0.85 (#5 Have you taken something from a store without paying for it in the past year? & #6 Have you stolen or tried to steal things worth $5 or less in the past year?).

### Excluded items for parent-report analyses

#### Full sample parent-report analyses (n=11,185)

A total of 131 items were included in analyses from three parent-report instruments. Five CBCL items were excluded due to low endorsement (q02 alcohol, q101 truancy, q105 drugs, q73 sexual problems, q99 tobacco). Two composite measures were created from 4 CBCL items (distracted: q08 can’t concentrate and q78 inattentive or easily distracted; destroys: q21 destroys things belonging to family and q20 destroys their own things) due to polychoric correlations >0.85.

#### Split sample parent-report analyses (first half=5590, second half = 5595)

A total of 126 items were included in analyses from three instruments. Twelve CBCL items (q02 alcohol, q101 truancy, q105 drugs, q73 sexual problems, q99 tobacco, q67 runs away from home, q72 sets fires, q96 thinks about sex too much, q110 wishes to be of opposite sex, q18 harms self, q98 thumb sucking) were excluded due to low endorsement that led to conflicting correlations between items. Two composite measures were created from 4 CBCL items (distracted: q08 can’t concentrate and q78 inattentive or easily distracted; destroys: q21 destroys things belonging to family and q20 destroys their own things) due to polychoric correlations >0.85.

### Conceptually-Based Model for Full Sample Analyses

For the full sample child-report analysis, we hypothesized that a bifactor model including four specific uncorrelated dimensions *(externalizing, internalizing, thought* and *impulse*), as well as a *general p* factor would fit the data well (Figure 1A). *A priori* target loading of items on their factors was determined based on previous factor analytic studies as discussed below. UPPS-P lack of premeditation and lack of perseverance measures were specified as loading onto an *externalizing* factor [(Watts et al., 2020; Zapolski et al., 2010; Zapolski & Smith, 2013)](https://paperpile.com/c/QBFBNP/bWbSY+Q1FDI+V8NLw) with BPM items corresponding to the CBCL’s aggressive and attention subscales, together with all items from the Delinquency Scale, and items from the Positive Affective Scale. Loading of Positive Affective items onto the *externalizing* factor were informed by a previous factor analysis in the NIH emotional toolbox that found that positive affect loaded with reverse-coded anger and separately from anxiety, sadness, and social withdrawal measures on a psychological well-being factor [(Paolillo et al., 2020)](https://paperpile.com/c/QBFBNP/ub1Dw). Urgency and sensation seeking items from the UPPS-P were specified as loading onto a specific *impulse* factor together with the BAS fun-seeking and drive items [(Cooper et al., 2007; Kingsbury et al., 2013)](https://paperpile.com/c/QBFBNP/7CC5C+BTXMB). These items have previously been shown to correlate highly [(Watts et al., 2020)](https://paperpile.com/c/QBFBNP/bWbSY) and are not strongly associated with externalizing items as they relate to reward sensitivity and novelty seeking. The *internalizing* factor was defined by items from the BPM corresponding to the CBCL anxious/depressed and withdrawn/depressed subscales and BIS inhibition/punishment sensitivity and BAS reward sensitivity items, as they have been shown to correlate with anxiety and depression [(Katz et al., 2020; Kingsbury et al., 2013)](https://paperpile.com/c/QBFBNP/BTXMB+Jjt2L). Although it is not entirely clear how PLEs fit into current structures of developmental psychopathology, there is evidence that they load with mania symptoms [(Cowan & Mittal, 2020; Keyes et al., 2013; Vaidyanathan et al., 2012)](https://paperpile.com/c/QBFBNP/hmXUT+UWBOm+yKBvW). Accordingly, all PQ-BC items were loaded onto a *thought/mania* factor along with all items from the Mania 7-UP Scale.

For the full sample parent-report analysis, we hypothesized that a bifactor model including four specific uncorrelated dimensions of psychopathology (*externalizing, internalizing, neurodevelopmental* and *thought*), as well as a *general p* factor would fit the data well (Figure 2A) based on previously reported empirical work using similar measures of psychopathology symptoms [(Afzali et al., 2018; Caspi et al., 2014; Haltigan et al., 2018; Laceulle et al., 2015; Michelini et al., 2019; Moore et al., 2020)](https://paperpile.com/c/QBFBNP/wvrE8+NLK5w+oJwcq+hYnJs+ozWi2+hvekj). Multiple factor analyses using the CBCL have identified a *general p* factor [(Haltigan et al., 2018; Michelini et al., 2019)](https://paperpile.com/c/QBFBNP/oJwcq+NLK5w), in addition to the specific broad-band *internalizing* and *externalizing* factors and eight syndrome subscales originally identified [(Achenbach, 1991; Achenbach et al., 2011)](https://paperpile.com/c/QBFBNP/kJUCp+XON6p). Another study using Goldberg’s “bass-ackwards” hierarchical method [(Goldberg, 2006)](https://paperpile.com/c/QBFBNP/90cvb) and CBCL items identified a higher-order *general p* factor and five lower-order factors: *internalizing, externalizing, somatoform, neurodevelopmental*, and *detachment* [(Michelini et al., 2019)](https://paperpile.com/c/QBFBNP/oJwcq). Other studies have also found separate specific *attention* and/or *neurodevelopmental* [(Bloemen et al., 2018; Moore et al., 2020; Noordhof et al., 2015)](https://paperpile.com/c/QBFBNP/wvrE8+qlzEH+ilbi9) and *thought* factors [(Haltigan et al., 2018)](https://paperpile.com/c/QBFBNP/NLK5w). Adding Mania and SRS items provided the advantage of more precisely disentangling their relationships with *thought* and *neurodevelopmental* factors, respectively. CBCL items were divided between the *externalizing* and *internalizing* factors as they have been previously [(Achenbach, 1991; Haltigan et al., 2018; Michelini et al., 2019; Moore et al., 2020)](https://paperpile.com/c/QBFBNP/XON6p+oJwcq+NLK5w+wvrE8). A separate *neurodevelopmental* factor with CBCL attention and previously identified neurodevelopmental items, as well as SRS items, were included based on prior evidence that it is a distinguishable specific factor [(Bloemen et al., 2018; Michelini et al., 2019; Noordhof et al., 2015)](https://paperpile.com/c/QBFBNP/qlzEH+ilbi9+oJwcq). Mania items and thought items from the CBCL were included in the *thought* factor [(Caspi et al., 2014; Cowan & Mittal, 2020; Keyes et al., 2013; Kotov et al., 2011; Vaidyanathan et al., 2012)](https://paperpile.com/c/QBFBNP/hmXUT+UWBOm+yKBvW+hYnJs+KWiCj).

##

## Results

### Child-report split sample ESEMs in the first half of the sample

A five-factor (RMSEA=0.018, CFI=0.955, TLI=0.948) ESEM showed the best fit in the first half of the sample for child-report items compared to models with fewer factors. This initial exploratory model included a 30-item *externalizing* factor (most delinquency, aggressive, attention, depression, and positive affective items), a 9-item *internalizing* factor (anxious/depressed and sensitivity to punishment items), an 11-item *reward sensitivity* factor (reward responsiveness, drive, fun seeking items), an 8-item *impulse* factor (positive and negative urgency items), and a 7-item *mania* factor (mania items). See Supplementary Table 2 for detailed loadings.

The four-factor model (RMSEA=0.021, CFI=0.941, TLI=0.935) was similar to the five-factor model, but did not have a *mania* factor. It included a 31-item *externalizing* factor (most delinquency, mania, attention and aggressive items), a 6-item *internalizing* factor (sensitivity to punishment items), a 15-item *reward sensitivity* factor (reward responsiveness, fun seeking, sensation seeking, and lack of perseverance items), and a 17-item *impulse* factor (most impulsivity items).

### Parent-report split sample ESEM in the first half of the sample

A five-factor model (RMSEA=0.015, CFI=0.963, TLI=0.960) ESEM in the first half of the sample showed the best fit for parent-report items compared to models with fewer factors. This model included a 40-item *externalizing* factor (predominantly aggressive, delinquent, and attention items), a 22-item *internalizing* factor (predominantly anxious/depressed items), a 31-item *neurodevelopmental* factor (neurodevelopmental, attention, thought, mania, and social communication items), a 16-item *social impairment* factor (social problems, withdrawn/depressed and social responsiveness items), and a 10-item *thought/somatic* factor (thought and somatic items).

The four-factor (RMSEA=0.016, CFI=0.958, TLI=0.955) included 41-item *externalizing* factor (predominantly aggressive, delinquent, attention, and mania items), a 33-item *internalizing* factor (anxious/depressed, withdrawn/depressed, somatic, and mania mood items), a 34-item neurodevelopmental items (neurodevelopmental, attention, anxious/depressed, and thought items), and a 17-item *social impairment* factor (social, withdrawn/depressed, and social responsiveness items).

### Adjustments to *a priori* factor loadings in child-report full sample B-ESEM

BAS items related to reward sensitivity were initially loaded onto the *internalizing* factor as they have been associated with social anxiety, and previous studies have linked reduced response to rewards to high risk for developing affective disorders [(Kingsbury et al., 2013)](https://paperpile.com/c/QBFBNP/BTXMB). However, BAS measures in general are more consistently associated with externalizing symptoms, while BIS inhibition items are associated with internalizing symptoms [(Colder & O’Connor, 2004)](https://paperpile.com/c/QBFBNP/XC3nt). In the initial model, factor loadings on the BPM anxiety/depression items for the *internalizing* factor were low (average internalizing BPM factor loading: 0.01), while BAS and BIS all showed high loadings on the *internalizing* factor. Thus, for the final model BAS reward sensitivity items were re-targeted to the *externalizing* factor with the aim of defining a clearer *internalizing* factor (average internalizing BPM factor loading: 0.29). Note that goodness of fit did not change between models as rotation does not impact these indices.

### Child-report full sample B-ESEM follow up analysis

A follow up ESEM analysis excluding a *general p* factor was run to investigate if PLEs would load more highly onto the *thought* factor. This model showed a poorer fit (RMSEA=0.021, CFI=0.939, TLI=0.933) than the B-ESEM model, but PLEs loaded more highly onto the *mania* factor, including three items with loadings ≥0.32 (sees things, believes they have unusual powers, and believes they have special abilities).

### External validation measures

General and specific factors defined by B-ESEMs using both child-report and parent-report models explained significant unique variance for multiple external measures as shown by simultaneous regressions in latent space within each SEM model (Table 1 & 2). In addition to findings of differences related to the *general p* factor detailed in the Results, the *externalizing* factor was associated with lower surface area, but higher cortical volume across all models. *Externalizing* factors were also associated with increased medical service usage in the child-report models. *Internalizing* factors were associated with elevated fluid and crystalized cognition in the parent-report models, but decreased fluid cognition in the child-report models. *Internalizing* factors were also associated with increased cortical volume and medical service usage in the parent-report models. The *mania* factor in the full sample child-report model was associated with decreased cognition, elevated surface area and reduced cortical volume, but less medical service usage. The *impulse* factors in the child-report models were associated with decreased crystalized cognition. The *reward sensitivity* factor in the child-report model was associated with higher cognition. The *neurodevelopmental* factors in the parent-report models were associated with decreased cognition and higher medical service usage. The *social impairment* factor was associated with higher crystalized cognition and lower fluid cognition and medical service usage. The *somatic* factor was associated with lower crystalized cognition, but higher fluid cognition and medical service usage.

### Correlations between factor scores across models

Pearson correlations of estimated psychopathology factor scores saved from each full sample model and the second half of the split sample B-ESEM approach showed that *general p, externalizing* and *internalizing* factors were highly correlated between the child-report full sample and child-report split sample models (p=0.89, EXT=0.62, INT=0.95) and from the parent-report full sample and parent-report split sample models (p=0.98, EXT=0.91, INT=0.87). However, correlations between the child-report and parent-report full sample models (p=0.27, EXT=0.05, INT=0.11), child-report and parent-report split sample models (p=0.24, EXT=0.08, INT=0.08), child-report full sample and parent-report split sample (p=0.26, EXT=0.08, INT=0.12), as well as the child-report split sample and parent-report full sample models (p=0.25, EXT=0.11, INT=0.08), were low to moderate.

#### Table S1. Psychopathology instruments included from the ABCD Study.

| **Informant** | **Scale** | **Timepoint** | **Description** | **Type of symptoms** |
| --- | --- | --- | --- | --- |
| Parent | Child Behavior Checklist (CBCL) | Baseline,  and then  annually | 119 questions | Anxious/depressed, withdrawn-depressed,  somatic complaints, delinquent, aggressive behavior, social and  thought syndrome scales |
| Parent | Mania Scale – Parent General Behavior Inventory | Baseline,  and then  annually | 10 questions | Mania |
| Parent | Social Responsiveness Scale | 1 year, every two years | 11 questions | ASD social communication and repetitive,  restrictive behavior |
| Youth | Prodromal  Questionnaire Brief Version | Baseline,  and then annually | 21 questions,  binary with a distress scale  of 1-5 | Positive psychotic-like experiences |
| Youth | Modified UPPS-P for Children from PhenX | Baseline, and then every  two years | 20 questions | Impulsivity (positive and negative urgency, lack of perseverance, lack of premeditation,  and sensation seeking) |
| Youth | Youth Behavioral Inhibition/Behavioral Approach System  Scales Modified from PhenX (BIS/BAS) | Baseline, and then every  two years | 20 questions | Behavioral Inhibition & Activation – (drive,  reward responsiveness,  fun seeking, and inhibition) |
| Youth | Positive Affective Items from the NIH Toolbox Battery | 6 months, 1 year | 9 questions | Positive emotions and affective well-being |
| Youth | Brief Problem Monitor | 6 months, 1 year | 19 questions | Anxious/depressed, withdrawn-depressed, attention, and aggressive  behavior |
| Youth | Mania – 7-Up | 1 year, every two years | 7 questions | Mania |
| Youth | Delinquency Scale | 1 year, every two years | 10 questions | Delinquency |

#### Table S2. Child-report exploratory ESEM in the first half of the sample and validation B-ESEM in the second half of the sample

|  |  | **Five-factor child-report exploratory ESEM** | | | | | **Four-factor child-report validation B-ESEM** | | | | |
| --- | --- | --- | --- | --- | --- | --- | --- | --- | --- | --- | --- |
| **Items** | **Description** | **INT** | **EXT** | **BAS** | **IMP** | **MAN** | **INT** | **EXT** | **BAS** | **IMP** | **P** |
| BISBAS1 | tense when I think something unpleasant is going to happen | 0.25 | 0.07 | **0.32** | 0.14 | -0.06 | 0.3 | 0.01 | 0.27 | 0.1 | 0.23 |
| BISBAS2 | worry about mistakes | **0.49** | -0.03 | 0.26 | 0.11 | 0 | **0.53** | -0.08 | 0.18 | 0.03 | 0.25 |
| BISBAS3 | am hurt when I get scolded | **0.46** | 0.07 | 0.31 | 0.18 | -0.17 | **0.5** | -0.03 | 0.2 | 0.16 | 0.19 |
| BISBAS4 | feel upset when I think someone is mad at me | **0.49** | 0.02 | 0.31 | 0.16 | -0.11 | **0.5** | -0.07 | 0.21 | 0.1 | 0.21 |
| BISBAS6 | feel worried when I do poorly | **0.41** | 0.02 | 0.31 | 0.1 | -0.1 | **0.5** | -0.09 | 0.27 | 0.04 | 0.2 |
| BISBAS7 | am fearful compared to my friends | **0.37** | -0.07 | 0.02 | 0.19 | 0.1 | **0.35** | -0.09 | -0.06 | 0.07 | 0.25 |
| BISBAS8 | feel excited and full of every when I get something I want | 0.11 | 0.11 | **0.5** | 0.09 | -0.03 | 0.19 | 0.02 | **0.33** | 0.11 | 0.27 |
| BISBAS  10 | get thrilled when good things happen | 0.11 | 0.02 | **0.66** | -0.15 | 0.03 | 0.24 | -0.1 | **0.49** | -0.06 | 0.22 |
| BISBAS  9 | when I am doing well, like to keep going | 0.1 | -0.07 | **0.6** | -0.14 | 0.04 | 0.17 | -0.19 | **0.48** | -0.16 | 0.19 |
| BISBAS  11 | it would excite me to win a contest | 0.07 | 0.06 | **0.6** | -0.12 | -0.01 | 0.14 | -0.11 | **0.47** | -0.1 | 0.15 |
| BISBAS  12 | get excited when see an opportunity | 0.09 | 0.09 | **0.59** | 0.02 | -0.05 | 0.17 | -0.05 | **0.38** | -0.01 | 0.27 |
| BISBAS  13 | when I want something I go all the way | 0.05 | -0.02 | **0.34** | 0.13 | 0.06 | 0.01 | -0.09 | 0.21 | 0.05 | 0.23 |
| BISBAS  14 | I do everything to get what I want | 0.13 | -0.06 | 0.24 | 0.23 | 0.16 | 0.03 | -0.12 | 0.07 | 0.11 | **0.34** |
| BISBAS  15 | when I see an opportunity I go for it all the way | 0.02 | 0.04 | **0.35** | 0.18 | 0.04 | 0.04 | -0.08 | 0.2 | 0.12 | 0.29 |
| BISBAS  16 | nobody can stop me when I want something | -0.01 | 0.03 | 0.21 | 0.22 | 0.1 | -0.07 | -0.04 | 0.1 | 0.17 | 0.29 |
| BISBAS  17 | I often do things for no other reason than they might be fun | -0.12 | 0.09 | **0.37** | 0.29 | 0.05 | -0.02 | 0.05 | **0.32** | 0.25 | **0.33** |
| BISBAS  18 | I crave excitement and new sensations | -0.08 | -0.01 | **0.6** | 0.05 | 0.11 | 0.01 | -0.1 | **0.55** | 0.02 | 0.27 |
| BISBAS  19 | I am always willing to try something new | -0.12 | -0.04 | **0.59** | -0.02 | 0.12 | -0.03 | -0.16 | **0.51** | 0 | 0.25 |
| BISBAS  20 | spur of the moment | -0.16 | 0.04 | **0.4** | 0.28 | 0.03 | -0.05 | 0.01 | **0.36** | 0.24 | 0.31 |
| BISBAS  5R | not fearful or nervous even when bad things happen | 0.2 | 0.11 | -0.09 | -0.11 | -0.21 | 0.22 | 0.1 | -0.12 | -0.08 | -0.09 |
| DELQ_1 | hit someone | -0.15 | **0.49** | 0.05 | 0.05 | 0.1 | -0.09 | **0.35** | 0.04 | 0 | **0.33** |
| DELQ_2 | hidden weapon | -0.03 | 0.25 | 0.05 | 0.11 | 0.27 | -0.18 | **0.41** | 0.01 | -0.1 | 0.29 |
| DELQ_3 | thrown objects at people | -0.11 | **0.5** | 0.01 | -0.03 | 0.26 | -0.19 | 0.27 | 0 | 0.03 | **0.39** |
| DELQ_7 | destroy things | 0 | **0.46** | 0.03 | -0.03 | 0.25 | -0.1 | 0.26 | -0.08 | 0.01 | **0.36** |
| DELQ_9 | rowdy | -0.12 | **0.5** | 0 | 0.08 | 0.26 | -0.18 | 0.25 | -0.05 | -0.09 | **0.54** |
| DELQ_5_6 |  | -0.03 | 0.3 | 0 | 0.12 | 0.2 | -0.14 | 0.29 | 0.05 | -0.08 | **0.51** |
| SUP_1 | anxious or tense | 0.05 | 0.18 | 0.02 | 0.07 | **0.48** | -0.1 | -0.06 | -0.04 | -0.12 | **0.63** |
| SUP_2 | lot of excitement, did new things | -0.02 | -0.06 | 0.05 | 0.06 | **0.59** | -0.13 | -0.18 | -0.04 | -0.12 | **0.54** |
| SUP_3 | insomnia | 0.05 | 0.23 | 0 | 0.09 | **0.45** | -0.13 | 0.03 | -0.08 | -0.09 | **0.63** |
| SUP_4 | vivid sensations | 0.05 | 0.14 | 0.04 | 0.08 | **0.63** | -0.16 | -0.06 | -0.13 | -0.12 | **0.7** |
| SUP_5 | fast thinking, better than others | -0.05 | 0.07 | 0.08 | 0.02 | **0.61** | -0.11 | -0.02 | 0.05 | -0.15 | **0.45** |
| SUP_6 | grandiose | -0.05 | 0.09 | 0.01 | 0.08 | **0.65** | -0.11 | -0.01 | -0.05 | -0.11 | **0.53** |
| SUP_7 | rapid thoughts and ideas | 0.03 | 0.18 | 0.04 | 0.06 | **0.57** | -0.08 | 0.07 | -0.04 | -0.14 | **0.59** |
| POA_1 | attentive | -0.06 | -0.25 | 0.08 | -0.12 | 0.14 | -0.05 | -0.27 | 0.12 | -0.1 | -0.09 |
| POA_2 | delighted | -0.06 | -0.19 | 0.15 | -0.09 | 0.17 | -0.05 | -0.24 | 0.12 | -0.09 | 0.05 |
| POA_3 | calm | 0 | **-0.4** | 0.08 | -0.03 | 0.14 | 0.01 | **-0.39** | 0 | -0.04 | -0.08 |
| POA_4 | at ease | -0.04 | -**0.34** | 0.12 | -0.05 | 0.13 | -0.03 | **-0.33** | 0.04 | -0.05 | -0.03 |
| POA_5 | enthusiastic | -0.07 | -0.18 | 0.16 | -0.06 | 0.25 | -0.12 | -0.19 | 0.15 | -0.11 | 0.16 |
| POA_6 | interested | -0.01 | -0.2 | 0.14 | -0.08 | 0.25 | -0.08 | -0.26 | 0.15 | -0.12 | 0.05 |
| POA_7 | confident | -0.15 | -**0.33** | 0.18 | -0.06 | 0.28 | -0.15 | **-0.4** | 0.19 | -0.09 | 0.04 |
| POA_8 | energetic | -0.14 | -0.12 | 0.15 | -0.02 | 0.25 | -0.17 | -0.19 | 0.17 | -0.04 | 0.15 |
| POA_9 | concentrate | -0.02 | -**0.57** | 0.05 | -0.04 | 0.22 | -0.07 | **-0.55** | 0.1 | -0.06 | -0.17 |
| BPM_1 | acts young | 0.12 | **0.45** | 0 | 0.03 | 0.08 | 0.04 | 0.3 | -0.02 | 0.03 | **0.42** |
| BPM_2 | argues a lot | 0 | **0.63** | 0.04 | 0.01 | 0 | -0.02 | **0.43** | 0.01 | 0.05 | **0.43** |
| BPM_3 | fail to finish | 0.12 | **0.55** | -0.09 | 0.04 | -0.01 | 0.06 | **0.45** | -0.14 | 0.04 | **0.36** |
| BPM_4 | trouble concentrating | 0.09 | **0.76** | -0.02 | 0 | 0.01 | 0.1 | **0.61** | -0.12 | -0.03 | **0.51** |
| BPM_5 | trouble sitting still | 0.03 | **0.63** | 0.05 | -0.03 | 0.03 | 0.03 | **0.47** | 0.03 | -0.04 | **0.47** |
| BPM_6 | destroy things | -0.08 | **0.53** | 0.02 | 0.07 | 0.17 | -0.09 | **0.39** | -0.01 | -0.01 | **0.55** |
| BPM_7 | I disobey my parents | -0.04 | **0.65** | 0.05 | -0.02 | -0.03 | -0.09 | **0.46** | 0.04 | 0.03 | **0.33** |
| BPM_8 | I disobey at school | -0.09 | **0.59** | 0.03 | 0.07 | 0.12 | -0.18 | **0.44** | 0.02 | 0.05 | **0.44** |
| BPM_9 | I feel worthless or inferior | 0.3 | **0.47** | -0.08 | 0.02 | 0.21 | 0.23 | **0.4** | -0.13 | -0.11 | **0.49** |
| BPM_10 | I act without stopping to think | -0.07 | **0.68** | 0.1 | 0.04 | 0 | -0.04 | **0.54** | 0.07 | 0.07 | **0.43** |
| BPM_11 | I am too fearful | **0.33** | **0.42** | -0.08 | -0.08 | 0.19 | 0.24 | 0.28 | -0.15 | -0.13 | **0.47** |
| BPM_12 | I feel too guilty | 0.28 | **0.45** | -0.07 | -0.02 | 0.16 | 0.18 | 0.3 | -0.11 | -0.12 | **0.49** |
| BPM_13 | I am self-conscious | **0.36** | **0.42** | -0.07 | -0.05 | 0.12 | 0.29 | 0.29 | -0.09 | -0.1 | **0.38** |
| BPM_14 | I am inattentive | 0.09 | **0.76** | 0 | -0.02 | 0.04 | 0.07 | **0.56** | -0.03 | -0.03 | **0.52** |
| BPM_15 | I am stubborn | -0.07 | **0.59** | 0.09 | -0.09 | -0.05 | -0.04 | **0.42** | 0.11 | -0.06 | 0.27 |
| BPM_16 | hot temper | -0.01 | **0.64** | 0.06 | 0 | 0.04 | -0.01 | **0.45** | 0.03 | -0.02 | **0.44** |
| BPM_17 | threaten to hurt people | -0.14 | **0.62** | 0.04 | 0.03 | 0.18 | -0.08 | **0.46** | 0.03 | -0.04 | **0.5** |
| BPM_18 | I am unhappy | 0.27 | **0.42** | -0.11 | 0.05 | 0.2 | 0.2 | **0.37** | -0.17 | -0.07 | **0.45** |
| BPM_19 | I worry a lot | **0.38** | **0.36** | -0.09 | -0.05 | 0.29 | 0.3 | 0.2 | -0.15 | -0.14 | **0.53** |
| PSY_1 | confusing places | 0.14 | 0.11 | 0.1 | 0.14 | 0.15 | 0.1 | 0.02 | 0.01 | 0.04 | 0.3 |
| PSY_2 | strange sounds | 0.15 | 0.1 | 0.05 | 0.18 | 0.06 | 0.07 | 0.06 | 0.01 | 0.06 | **0.35** |
| PSY_3 | things appear different | 0.13 | 0.07 | 0.01 | 0.16 | 0.19 | 0.03 | -0.03 | 0 | 0.06 | **0.38** |
| PSY_4 | unusual powers | 0.02 | 0.11 | 0.18 | 0.11 | 0.27 | 0.03 | -0.01 | 0.07 | 0.04 | **0.35** |
| PSY_5 | someone in control | 0.21 | 0.09 | 0.01 | 0.2 | 0.21 | 0.13 | -0.02 | -0.03 | 0.04 | **0.4** |
| PSY_6 | speech difficulties | 0.22 | 0.06 | 0.06 | 0.18 | 0.11 | 0.13 | 0.01 | -0.04 | 0.05 | 0.31 |
| PSY_7 | special abilities | 0 | 0.05 | 0.11 | 0.15 | 0.32 | 0.01 | -0.05 | 0.09 | 0.07 | **0.38** |
| PSY_8 | couldn't trust | 0.23 | 0.02 | 0.06 | 0.21 | 0.18 | 0.18 | -0.05 | -0.07 | 0.04 | 0.4 |
| PSY_9 | strange feelings | 0.18 | 0.14 | 0.04 | 0.15 | 0.13 | -0.01 | 0.04 | 0.02 | 0.03 | **0.36** |
| PSY_10 | lose concentration | 0.13 | 0.13 | 0.06 | 0.19 | 0.16 | 0.04 | 0.02 | 0.05 | 0.06 | **0.38** |
| PSY_11 | invisible energy | 0.17 | 0.21 | 0.13 | 0.1 | 0.09 | 0.09 | 0.12 | 0.07 | 0.03 | **0.34** |
| PSY_12 | mind is trying to trick you | 0.12 | 0.14 | 0.09 | 0.14 | 0.12 | 0.09 | 0.01 | 0.03 | 0.05 | **0.34** |
| PSY_13 | world not real | 0.13 | 0.14 | 0.08 | 0.19 | 0.19 | 0.17 | 0.17 | 0.1 | 0.16 | **0.33** |
| PSY_14 | feel confused | 0.12 | 0.1 | 0.09 | 0.18 | 0.21 | 0.07 | 0.03 | 0.07 | 0.06 | **0.36** |
| PSY_15 | strange beliefs | 0.13 | 0.13 | 0.11 | 0.09 | 0.09 | 0.04 | 0.03 | 0.09 | 0.02 | 0.28 |
| PSY_16 | body change | 0.1 | 0.04 | 0.05 | 0.18 | 0.27 | -0.01 | 0.01 | -0.01 | 0.04 | **0.41** |
| PSY_17 | hear thoughts | 0.06 | 0.15 | 0.09 | 0.09 | 0.18 | 0.07 | 0.01 | 0.15 | 0.04 | **0.32** |
| PSY_18 | suspicious | 0.18 | 0.14 | 0.08 | 0.16 | 0.16 | 0.13 | 0.04 | 0.02 | 0.08 | **0.36** |
| PSY_19 | unusual new sights | 0.11 | 0.13 | 0.04 | 0.17 | 0.2 | 0.03 | 0.06 | 0 | 0 | **0.38** |
| PSY_20 | see things | 0.06 | 0.1 | 0.11 | 0.18 | 0.26 | -0.01 | 0.04 | 0.04 | -0.02 | **0.43** |
| PSY_21 | disorganized speech | 0.14 | 0.09 | 0.05 | 0.16 | 0.21 | 0.05 | -0.05 | -0.05 | 0.09 | **0.39** |
| UPPS6 | stop and think | -0.26 | **0.35** | -0.04 | 0.28 | -0.21 | -0.19 | **0.35** | -0.02 | 0.3 | 0.07 |
| UPPS7 | do things I regret | 0.14 | -0.12 | 0.05 | **0.5** | 0.07 | 0.14 | -0.06 | 0.01 | **0.35** | 0.29 |
| UPPS11 | keep doing bad things | 0.09 | 0.03 | -0.06 | **0.55** | 0.02 | 0.06 | 0.09 | -0.04 | **0.46** | 0.28 |
| UPPS12 | enjoy taking risks | -0.31 | 0.12 | 0.27 | 0.23 | -0.02 | -0.21 | 0.1 | **0.34** | 0.22 | 0.1 |
| UPPS15 | finish what I start | 0.06 | 0.12 | -0.25 | 0.27 | -0.11 | 0.04 | 0.17 | -0.28 | 0.22 | 0.02 |
| UPPS16 | take a careful approach to things | -0.3 | 0.24 | -0.11 | 0.2 | -0.13 | -0.26 | 0.24 | -0.04 | 0.23 | -0.01 |
| UPPS17 | when I am upset I act without thinking | 0.05 | 0.23 | 0.12 | **0.43** | -0.18 | 0.15 | 0.24 | 0.15 | **0.46** | 0.19 |
| UPPS18 | I like new thrilling things | -0.24 | 0.04 | **0.39** | 0.15 | -0.01 | -0.16 | 0.04 | **0.37** | 0.12 | 0.14 |
| UPPS19 | I tend to get things done on time | 0.04 | 0.26 | -0.17 | 0.29 | -0.1 | -0.02 | 0.28 | -0.18 | 0.26 | 0.1 |
| UPPS20 | when rejected say things they regret | 0.14 | 0.03 | 0.08 | **0.55** | -0.06 | 0.14 | 0.08 | 0.04 | **0.47** | 0.24 |
| UPPS21 | fly an airplane | -0.12 | -0.1 | 0.08 | 0.08 | 0.14 | -0.11 | -0.1 | 0.14 | 0.05 | 0.06 |
| UPPS22 | gets the job done | 0.05 | 0.24 | -0.28 | 0.26 | -0.16 | 0.03 | 0.29 | -0.27 | 0.29 | 0.04 |
| UPPS23 | very careful | -0.32 | **0.33** | -0.01 | 0.24 | -0.18 | -0.27 | 0.31 | 0.09 | 0.3 | 0.04 |
| UPPS24 | always finish projects that I start | 0.02 | 0.14 | -0.27 | 0.26 | -0.04 | -0.02 | 0.2 | -0.27 | 0.23 | 0.09 |
| UPPS27 | ski fast | -0.29 | -0.02 | 0.25 | 0.11 | 0.05 | -0.24 | -0.02 | 0.3 | 0.12 | 0.04 |
| UPPS28 | stop and think before doing | -0.3 | **0.39** | -0.02 | 0.31 | -0.29 | -0.2 | **0.4** | 0.04 | **0.35** | 0.04 |
| UPPS35 | when I am in a great mood I do things that cause problems | -0.02 | -0.04 | -0.08 | **0.72** | 0.01 | -0.04 | 0.09 | -0.01 | **0.65** | 0.25 |
| UPPS36 | act without thinking when I am very happy | 0.02 | -0.07 | 0.01 | **0.7** | 0.14 | -0.04 | -0.05 | 0.08 | **0.56** | **0.42** |
| UPPS37 | when happy do bad things | -0.04 | -0.02 | -0.04 | **0.81** | 0.04 | -0.07 | 0.05 | 0.01 | **0.7** | **0.35** |
| UPPS39 | I lost control when in great mood | 0.02 | -0.03 | 0.04 | **0.68** | 0.1 | -0.04 | -0.04 | 0.02 | **0.57** | **0.44** |

#### Table S3. Child-report full sample B-ESEM

|  |  | **Four-factor child-report full sample B-ESEM** | | | | |
| --- | --- | --- | --- | --- | --- | --- |
| **Items** | **Description** | **EXT** | **INT** | **MAN** | **IMP** | **P** |
| BISBAS1 | tense for something unpleasant | -0.17 | 0.24 | -0.13 | 0.14 | **0.32** |
| BISBAS10 | get thrilled when good things happen | **-0.47** | 0.08 | -0.21 | 0.00 | **0.37** |
| BISBAS11 | excited to win a contest | **-0.41** | 0.02 | -0.21 | 0.00 | **0.32** |
| BISBAS12 | get excited, see an opportunity | **-0.32** | 0.06 | -0.18 | 0.09 | **0.38** |
| BISBAS13 | want something, go all the way | -0.20 | -0.01 | -0.01 | 0.14 | 0.26 |
| BISBAS14 | I do everything to get what I want | -0.14 | 0.05 | 0.14 | 0.19 | 0.29 |
| BISBAS15 | see an opportunity go for it | -0.16 | 0.00 | -0.01 | 0.19 | **0.31** |
| BISBAS16 | nobody can stop me, want something | -0.06 | -0.05 | 0.07 | 0.21 | 0.27 |
| BISBAS17 | do things as they might be fun | -0.10 | -0.10 | -0.09 | 0.31 | **0.40** |
| BISBAS18 | crave excitement and new sensations | **-0.41** | -0.12 | -0.16 | 0.14 | **0.41** |
| BISBAS19 | always willing to try something new | **-0.43** | -0.16 | -0.12 | 0.11 | **0.35** |
| BISBAS2 | worry about mistakes | -0.25 | **0.46** | -0.04 | 0.07 | 0.30 |
| BISBAS20 | spur of the moment | -0.15 | -0.14 | -0.11 | **0.32** | **0.37** |
| BISBAS3 | am hurt when I get scolded | -0.18 | **0.46** | -0.16 | 0.17 | 0.28 |
| BISBAS4 | upset when someone is mad | -0.23 | **0.45** | -0.12 | 0.14 | 0.29 |
| BISBAS5R | not fearful or nervous bad things | 0.10 | 0.24 | -0.09 | -0.13 | -0.09 |
| BISBAS6 | feel worried when I do poorly | -0.26 | **0.40** | -0.13 | 0.10 | 0.28 |
| BISBAS7 | am fearful compared to my friends | -0.08 | **0.35** | 0.14 | 0.10 | 0.18 |
| BISBAS8 | excited and energy, something I want | -0.23 | 0.10 | -0.16 | 0.16 | **0.39** |
| BISBAS9 | doing well, like to keep going | **-0.51** | 0.03 | -0.15 | -0.03 | **0.30** |
| BPM_1 | acts young | 0.25 | 0.10 | 0.05 | -0.02 | **0.45** |
| BPM_10 | I act without stopping to think | **0.38** | -0.03 | -0.11 | 0.00 | **0.57** |
| BPM_11 | I am too fearful | 0.22 | 0.29 | 0.16 | -0.19 | **0.46** |
| BPM_12 | I feel too guilty | 0.24 | 0.24 | 0.14 | -0.14 | **0.48** |
| BPM_13 | I am self-conscious | 0.21 | **0.33** | 0.08 | -0.16 | **0.42** |
| BPM_14 | I am inattentive | **0.43** | 0.11 | -0.02 | -0.11 | **0.63** |
| BPM_15 | I am stubborn | 0.28 | -0.05 | -0.14 | -0.10 | **0.42** |
| BPM_16 | hot temper | **0.34** | 0.01 | -0.04 | -0.06 | **0.55** |
| BPM_17 | threaten to hurt people | **0.35** | -0.10 | 0.04 | -0.06 | **0.59** |
| BPM_18 | I am unhappy | **0.30** | 0.25 | 0.16 | -0.11 | **0.47** |
| BPM_19 | I worry a lot | 0.16 | **0.34** | 0.24 | -0.18 | **0.49** |
| BPM_2 | argues a lot | **0.35** | 0.01 | -0.04 | -0.02 | **0.51** |
| BPM_3 | fail to finish | **0.41** | 0.13 | 0.01 | -0.05 | **0.41** |
| BPM_4 | trouble concentrating | **0.49** | 0.13 | -0.02 | -0.11 | **0.60** |
| BPM_5 | trouble sitting still | **0.33** | 0.04 | -0.03 | -0.09 | **0.55** |
| BPM_6 | destroy things | **0.32** | -0.06 | 0.09 | -0.02 | **0.57** |
| BPM_7 | I disobey my parents | **0.36** | -0.04 | -0.10 | -0.05 | **0.47** |
| BPM_8 | I disobey at school | **0.36** | -0.11 | 0.02 | 0.00 | **0.53** |
| BPM_9 | I feel worthless or inferior | 0.29 | 0.27 | 0.14 | -0.15 | **0.53** |
| DELQ_1 | hit someone | 0.27 | -0.10 | -0.02 | -0.01 | **0.43** |
| DELQ_2 | hidden weapon | 0.20 | -0.11 | 0.09 | -0.05 | **0.41** |
| DELQ_3 | thrown objects at people | 0.26 | -0.14 | 0.12 | -0.04 | **0.46** |
| DELQ_4 | gang fight | 0.29 | -0.09 | 0.27 | 0.02 | **0.39** |
| DELQ_5_6 | stolen | 0.22 | -0.05 | 0.13 | 0.02 | **0.38** |
| DELQ_7 | destroy things | 0.22 | -0.08 | 0.18 | -0.09 | **0.52** |
| DELQ_9 | rowdy | 0.25 | -0.12 | 0.12 | -0.04 | **0.55** |
| POA_1 | attentive | -0.27 | -0.09 | 0.05 | -0.05 | -0.12 |
| POA_2 | delighted | -0.26 | -0.09 | 0.07 | -0.03 | 0.01 |
| POA_3 | calm | **-0.32** | -0.02 | 0.13 | 0.02 | -0.18 |
| POA_4 | at ease | **-0.30** | -0.06 | 0.10 | 0.01 | -0.12 |
| POA_5 | enthusiastic | -0.25 | -0.14 | 0.12 | -0.03 | 0.10 |
| POA_6 | interested | -0.29 | -0.09 | 0.12 | -0.05 | 0.04 |
| POA_7 | confident | **-0.38** | -0.20 | 0.14 | 0.02 | -0.03 |
| POA_8 | energetic | -0.21 | -0.19 | 0.10 | 0.02 | 0.12 |
| POA_9 | concentrate | **-0.45** | -0.09 | 0.16 | 0.02 | -0.29 |
| PSY_1 | confusing places | -0.01 | 0.11 | 0.10 | 0.07 | **0.30** |
| PSY_10 | lose concentration | 0.02 | 0.08 | 0.13 | 0.11 | **0.35** |
| PSY_11 | invisible energy | 0.03 | 0.12 | 0.04 | 0.05 | **0.37** |
| PSY_12 | mind is trying to trick you | 0.00 | 0.10 | 0.09 | 0.09 | **0.32** |
| PSY_13 | world not real | 0.06 | 0.13 | 0.06 | 0.14 | **0.38** |
| PSY_14 | feel confused | -0.01 | 0.08 | 0.12 | 0.10 | **0.36** |
| PSY_15 | strange beliefs | -0.02 | 0.07 | 0.05 | 0.05 | 0.29 |
| PSY_16 | body change | 0.00 | 0.04 | 0.21 | 0.09 | **0.34** |
| PSY_17 | hear thoughts | -0.03 | 0.05 | 0.07 | 0.06 | **0.34** |
| PSY_18 | suspicious | 0.02 | 0.15 | 0.11 | 0.09 | **0.35** |
| PSY_19 | unusual new sights | 0.04 | 0.06 | 0.15 | 0.06 | **0.35** |
| PSY_2 | strange sounds | 0.04 | 0.12 | 0.09 | 0.10 | 0.29 |
| PSY_20 | see things | -0.01 | 0.02 | 0.17 | 0.07 | **0.40** |
| PSY_21 | disorganized speech | 0.01 | 0.10 | 0.20 | 0.11 | **0.31** |
| PSY_3 | things appear different | 0.01 | 0.08 | 0.18 | 0.10 | **0.30** |
| PSY_4 | unusual powers | -0.06 | 0.01 | 0.14 | 0.08 | **0.37** |
| PSY_5 | someone in control | 0.02 | 0.17 | 0.19 | 0.09 | **0.34** |
| PSY_6 | speech difficulties | 0.01 | 0.17 | 0.12 | 0.09 | 0.27 |
| PSY_7 | special abilities | -0.07 | -0.02 | 0.18 | 0.11 | **0.35** |
| PSY_8 | couldn't trust | -0.03 | 0.20 | 0.19 | 0.10 | **0.31** |
| PSY_9 | strange feelings | 0.04 | 0.09 | 0.13 | 0.07 | **0.33** |
| SUP_1 | anxious or tense | 0.00 | -0.03 | **0.35** | -0.04 | **0.50** |
| SUP_2 | lot of excitement, did new things | -0.14 | -0.10 | **0.41** | -0.02 | **0.38** |
| SUP_3 | insomnia | 0.08 | -0.04 | **0.34** | -0.03 | **0.52** |
| SUP_4 | vivid sensations | 0.00 | -0.06 | **0.47** | -0.04 | **0.55** |
| SUP_5 | fast thinking, better than others | -0.07 | -0.11 | 0.29 | -0.07 | **0.44** |
| SUP_6 | grandiose | -0.01 | -0.09 | **0.37** | -0.04 | **0.47** |
| SUP_7 | rapid thoughts and ideas | 0.03 | -0.04 | **0.33** | -0.07 | **0.53** |
| UPPS11 | keep doing bad things | 0.19 | 0.12 | 0.08 | **0.45** | 0.23 |
| UPPS12 | enjoy taking risks | -0.02 | -0.28 | -0.17 | 0.28 | 0.23 |
| UPPS15 | finish what I start | **0.33** | 0.12 | 0.06 | 0.16 | -0.02 |
| UPPS16 | take a careful approach to things | **0.33** | -0.23 | -0.09 | 0.18 | 0.03 |
| UPPS17 | when I am upset I act without thinking | 0.19 | 0.12 | -0.18 | **0.41** | **0.30** |
| UPPS18 | I like new thrilling things | -0.15 | -0.24 | -0.18 | 0.21 | 0.25 |
| UPPS19 | I tend to get things done on time | **0.37** | 0.08 | 0.00 | 0.19 | 0.13 |
| UPPS20 | when rejected say things they regret | 0.11 | 0.17 | -0.02 | **0.47** | 0.26 |
| UPPS21 | fly an airplane | -0.12 | -0.14 | 0.05 | 0.09 | 0.06 |
| UPPS22 | gets the job done | **0.44** | 0.12 | 0.00 | 0.18 | 0.02 |
| UPPS23 | very careful | **0.33** | -0.25 | -0.18 | 0.25 | 0.14 |
| UPPS24 | always finish projects that I start | **0.36** | 0.07 | 0.10 | 0.16 | 0.03 |
| UPPS27 | ski fast | -0.12 | -0.29 | -0.10 | 0.18 | 0.12 |
| UPPS28 | stop and think before doing | **0.42** | -0.19 | -0.24 | 0.29 | 0.15 |
| UPPS35 | great mood, cause problems | 0.23 | 0.03 | 0.06 | **0.63** | 0.20 |
| UPPS36 | act without thinking, very happy | 0.09 | 0.02 | 0.16 | **0.60** | **0.33** |
| UPPS37 | when happy do bad things | 0.22 | 0.00 | 0.10 | **0.70** | 0.28 |
| UPPS39 | I lost control when in great mood | 0.11 | 0.03 | 0.16 | **0.60** | **0.33** |
| UPPS6 | stop and think | **0.39** | -0.16 | -0.17 | 0.25 | 0.14 |
| UPPS7 | do things I regret | 0.00 | 0.16 | 0.11 | **0.40** | 0.22 |

#### Table S4. Parent-report exploratory ESEM in the first half of the sample and validation B-ESEM in the second half of the sample

|  |  | **Parent-Report Exploratory Five-factor ESEM** | | | | | **Parent-Report Validation Five-factor**  **B-ESEM** | | | | | |
| --- | --- | --- | --- | --- | --- | --- | --- | --- | --- | --- | --- | --- |
| **Items** | **Description** | **EXT** | **INT** | **NDD** | **SOC** | **SOM** | **EXT** | **INT** | **NDD** | **SOC** | **SOM** | **P** |
| CBCL_01 | Too young | 0.12 | 0 | **0.45** | 0.29 | -0.09 | 0.1 | -0.02 | 0.29 | 0.17 | -0.07 | **0.56** |
| CBCL_03 | argues | **0.71** | 0.24 | 0.07 | -0.07 | -0.05 | **0.48** | 0.12 | -0.03 | -0.12 | -0.05 | **0.63** |
| CBCL_04 | fails to finish | 0.22 | 0.01 | **0.55** | 0.08 | 0.04 | 0.15 | -0.1 | 0.26 | -0.01 | 0 | **0.64** |
| CBCL_05 | little they enjoy | 0.16 | 0.22 | 0.05 | **0.41** | 0.19 | 0.1 | -0.01 | -0.1 | 0.09 | -0.16 | **0.68** |
| CBCL_06 | bowel movements | **0.5** | 0.09 | 0.24 | -0.21 | -0.05 | **0.43** | 0.17 | 0.21 | -0.08 | 0.04 | **0.35** |
| CBCL_07 | bragging | 0.06 | 0.31 | **0.52** | 0.06 | 0.01 | 0 | 0.11 | 0.27 | 0.03 | -0.01 | **0.71** |
| CBCL_09 | obsessions | 0.15 | 0.07 | **0.78** | -0.13 | -0.01 | 0.16 | -0.13 | **0.51** | -0.15 | 0.04 | **0.64** |
| CBCL_10 | restless | 0.08 | 0.28 | **0.33** | 0.05 | 0.09 | -0.07 | 0.11 | 0.08 | -0.07 | -0.02 | **0.64** |
| CBCL_11 | clings to people | 0.18 | **0.34** | 0.2 | 0.26 | -0.06 | 0.01 | 0.27 | 0.09 | 0.1 | -0.11 | **0.64** |
| CBCL_12 | lonely | -0.16 | 0.04 | **0.6** | 0.23 | 0.26 | -0.18 | -0.15 | 0.18 | 0.04 | -0.09 | **0.74** |
| CBCL_13 | confused | 0.22 | **0.35** | 0.15 | 0.08 | 0.06 | 0.04 | 0.17 | -0.04 | -0.1 | -0.14 | **0.63** |
| CBCL_14 | cries a lot | **0.45** | -0.14 | 0.12 | 0.2 | 0.17 | **0.4** | -0.01 | 0.03 | 0.02 | -0.09 | **0.54** |
| CBCL_15 | cruel to animals | **0.74** | 0.02 | -0.05 | 0.07 | 0.04 | **0.6** | -0.01 | -0.07 | 0.01 | -0.06 | **0.55** |
| CBCL_16 | cruel/bullies | -0.16 | 0.08 | **0.57** | 0.18 | 0.16 | -0.12 | -0.08 | 0.31 | 0.14 | 0.1 | **0.56** |
| CBCL_17 | daydreams | **0.35** | 0.25 | -0.02 | 0.3 | 0.1 | 0.15 | 0.31 | -0.05 | 0.02 | 0.05 | **0.54** |
| CBCL_19 | demands attention | **0.46** | 0.3 | **0.34** | -0.16 | -0.09 | 0.28 | 0.21 | 0.22 | -0.18 | -0.08 | **0.67** |
| CBCL_22 | disobedient at home | **0.83** | 0.07 | 0.02 | -0.05 | 0.04 | **0.6** | -0.06 | 0 | -0.05 | 0.07 | **0.61** |
| CBCL_23 | disobedient at school | **0.63** | -0.21 | 0.27 | 0.06 | 0.03 | **0.54** | -0.18 | 0.24 | 0.06 | -0.03 | **0.55** |
| CBCL_24 | doesn't eat well | 0.09 | 0.16 | 0.17 | 0.1 | 0.21 | 0.06 | 0.01 | 0.02 | 0.06 | 0.15 | **0.45** |
| CBCL_25 | doesn't get along well with others | **0.53** | 0.02 | 0.1 | **0.43** | -0.11 | **0.35** | 0.1 | 0.1 | **0.4** | -0.18 | **0.63** |
| CBCL_26 | doesn't feel guilty | **0.58** | -0.06 | 0.15 | 0.08 | 0.11 | **0.46** | -0.16 | 0.02 | -0.04 | -0.06 | **0.59** |
| CBCL_27 | easily jealous | **0.49** | 0.3 | 0.11 | -0.02 | -0.03 | 0.3 | 0.21 | -0.05 | -0.09 | -0.13 | **0.62** |
| CBCL_28 | breaks rules | **0.8** | -0.06 | 0.14 | -0.03 | 0.07 | **0.63** | -0.16 | 0.11 | -0.01 | 0.08 | **0.59** |
| CBCL_29 | fears animals | -0.01 | 0.31 | 0.22 | 0.04 | 0.13 | -0.14 | 0.19 | 0 | -0.02 | 0.19 | **0.49** |
| CBCL_30 | fears going to school | 0.06 | 0.31 | 0.09 | **0.33** | 0.14 | -0.08 | **0.34** | 0.01 | 0.2 | 0.05 | **0.55** |
| CBCL_31 | fears they will do bad | 0.04 | **0.48** | 0.19 | 0.12 | -0.04 | -0.06 | **0.4** | -0.02 | 0.04 | 0.1 | **0.53** |
| CBCL_32 | has to be perfect | -0.06 | **0.63** | 0.01 | 0.05 | -0.03 | -0.1 | **0.52** | -0.11 | 0.08 | 0.12 | **0.36** |
| CBCL_33 | feels no one loves them | **0.41** | **0.41** | -0.04 | 0.22 | -0.04 | 0.23 | **0.38** | -0.08 | 0.05 | -0.14 | **0.64** |
| CBCL_34 | others are out to get them | **0.35** | **0.35** | 0.07 | 0.27 | -0.05 | 0.23 | 0.24 | -0.02 | 0.1 | -0.11 | **0.66** |
| CBCL_35 | feels worthless | 0.19 | **0.48** | 0.07 | 0.31 | -0.07 | 0.09 | **0.47** | 0 | 0.18 | -0.01 | **0.62** |
| CBCL_36 | accident prone | 0.07 | 0.07 | **0.44** | -0.06 | 0.08 | 0.08 | 0.1 | 0.26 | 0.03 | 0.11 | **0.4** |
| CBCL_37 | gets into fights | **0.67** | -0.05 | 0.08 | 0.13 | 0 | **0.57** | -0.05 | 0.04 | 0.13 | -0.03 | **0.54** |
| CBCL_38 | gets teased | 0.27 | 0.03 | 0.24 | **0.44** | -0.12 | 0.11 | 0.18 | 0.22 | **0.35** | -0.16 | **0.54** |
| CBCL_39 | hangs out with trouble | **0.48** | -0.09 | 0.19 | -0.04 | 0.13 | 0.4 | -0.07 | 0.19 | 0.02 | -0.01 | **0.41** |
| CBCL_40 | hears sounds or voices | 0.19 | -0.17 | 0.15 | 0.14 | **0.57** | -0.04 | -0.14 | -0.01 | -0.08 | 0.19 | **0.61** |
| CBCL_41 | impulsive | **0.43** | 0.08 | **0.48** | 0.01 | -0.06 | **0.37** | -0.02 | **0.36** | 0.01 | 0.05 | **0.66** |
| CBCL_42 | rather be alone | -0.04 | 0.11 | -0.03 | **0.67** | 0.26 | -0.12 | -0.05 | -0.17 | **0.5** | 0.08 | **0.57** |
| CBCL_43 | lying or cheating | **0.65** | -0.12 | 0.15 | -0.04 | 0.13 | **0.5** | -0.13 | 0.11 | -0.01 | 0.04 | **0.53** |
| CBCL_44 | bites fingernails | 0.04 | 0.11 | 0.2 | 0.01 | 0.08 | 0.09 | 0.05 | 0.1 | 0.04 | 0.13 | 0.27 |
| CBCL_45 | nervous, tense | -0.04 | **0.57** | **0.34** | 0.03 | 0.11 | -0.04 | 0.25 | 0.09 | 0.01 | 0.24 | **0.7** |
| CBCL_46 | nervous movements | -0.13 | 0.27 | **0.54** | 0.03 | 0.11 | -0.01 | 0.03 | 0.27 | -0.01 | 0.17 | **0.6** |
| CBCL_47 | nightmares | 0.11 | 0.23 | 0.2 | -0.06 | 0.25 | 0.01 | 0.12 | 0.12 | -0.07 | 0.26 | **0.49** |
| CBCL_48 | not liked | **0.36** | 0 | 0.2 | **0.57** | -0.15 | 0.22 | 0.22 | 0.23 | **0.44** | -0.19 | **0.62** |
| CBCL_49 | constipated | 0.07 | 0.18 | 0.07 | 0.01 | 0.25 | -0.06 | 0.17 | 0.04 | 0.12 | 0.22 | **0.33** |
| CBCL_50 | too fearful or anxious | -0.14 | **0.64** | 0.27 | 0.09 | 0.08 | -0.2 | **0.35** | 0.03 | 0.05 | 0.26 | **0.66** |
| CBCL_51 | feels dizzy | 0.02 | 0.21 | 0.12 | -0.01 | **0.44** | -0.15 | 0.18 | -0.05 | -0.14 | 0.23 | **0.44** |
| CBCL_52 | feels too guilty | -0.02 | **0.57** | 0.18 | 0.07 | -0.01 | -0.11 | **0.48** | 0.02 | 0.05 | 0.11 | 0.57 |
| CBCL_53 | overeating | **0.43** | -0.19 | 0.16 | 0.16 | -0.2 | -0.08 | 0.16 | 0.02 | -0.05 | **-0.45** | 0.43 |
| CBCL_54 | overtired without reason | 0.17 | 0.09 | 0.18 | 0.2 | 0.22 | -0.12 | 0.03 | -0.12 | -0.03 | -0.06 | 0.62 |
| CBCL_55 | overweight | **0.33** | -0.24 | 0.06 | 0.2 | -0.24 | -0.11 | 0.1 | -0.02 | 0.02 | **-0.48** | 0.22 |
| CBCL_56A | aches | 0.07 | 0.16 | 0.09 | -0.11 | **0.33** | -0.01 | 0.17 | 0.03 | -0.05 | 0.27 | 0.32 |
| CBCL_56B | headaches | 0.09 | 0.13 | 0 | -0.1 | **0.42** | -0.02 | 0.16 | -0.04 | -0.12 | 0.28 | 0.32 |
| CBCL_56C | nausea | 0.08 | 0.23 | 0.02 | -0.19 | **0.56** | -0.04 | 0.24 | -0.01 | -0.13 | **0.43** | **0.37** |
| CBCL_56D | problems with eyes | -0.02 | 0.01 | 0.12 | 0.08 | **0.36** | -0.14 | -0.02 | -0.07 | -0.11 | 0.05 | **0.34** |
| CBCL_56E | rashes or skin problems | 0.09 | 0.06 | 0.13 | -0.08 | 0.21 | -0.04 | 0.09 | 0.03 | -0.05 | 0.14 | 0.27 |
| CBCL_56F | stomachaches | 0.1 | 0.23 | -0.01 | -0.23 | **0.55** | -0.03 | 0.25 | -0.02 | -0.1 | **0.38** | 0.32 |
| CBCL_56G | vomiting | 0.09 | 0.01 | 0.08 | -0.19 | **0.47** | 0.03 | 0.03 | 0.03 | -0.08 | **0.35** | 0.26 |
| CBCL_56H | somatoform problems | 0.16 | 0.15 | 0 | -0.01 | **0.37** | -0.1 | 0.08 | 0.11 | -0.07 | 0.25 | **0.38** |
| CBCL_57 | attacks people | **0.73** | 0.1 | -0.17 | 0.17 | 0 | **0.58** | -0.02 | -0.09 | 0.08 | 0.03 | **0.59** |
| CBCL_58 | picks nose, skin | 0.18 | 0.13 | 0.24 | 0.06 | 0.03 | 0.16 | 0.07 | 0.24 | 0.18 | 0.22 | **0.38** |
| CBCL_60 | plays with sex parts too much | 0.26 | 0.02 | 0.18 | -0.05 | 0.06 | 0.24 | 0.01 | 0.24 | 0.16 | 0.04 | **0.36** |
| CBCL_61 | poor schoolwork | 0.24 | -0.14 | **0.49** | 0.16 | 0.09 | 0.21 | -0.19 | 0.29 | 0.14 | -0.07 | **0.58** |
| CBCL_62 | poor coordination | -0.01 | -0.04 | **0.6** | 0.16 | 0.06 | -0.04 | 0.04 | **0.34** | 0.2 | 0.03 | **0.56** |
| CBCL_63 | prefers older kids | 0.26 | 0.03 | 0.21 | 0.03 | 0.09 | 0.16 | 0.02 | 0.11 | 0.02 | 0.01 | **0.43** |
| CBCL_64 | prefers younger kids | 0.1 | 0.05 | **0.34** | 0.2 | 0.03 | -0.01 | 0.01 | 0.15 | 0.15 | -0.02 | **0.51** |
| CBCL_65 | refuses to talk | 0.18 | 0.13 | -0.02 | **0.34** | 0.32 | -0.03 | -0.11 | -0.27 | 0.19 | 0.05 | **0.66** |
| CBCL_66 | repeats acts | 0.06 | 0.11 | **0.49** | 0.11 | 0.15 | 0 | -0.09 | 0.19 | 0.07 | -0.04 | **0.74** |
| CBCL_68 | screams a lot | **0.59** | 0.22 | 0.04 | 0.06 | 0.02 | **0.39** | 0.04 | -0.08 | -0.15 | -0.11 | **0.66** |
| CBCL_69 | secretive | 0.23 | 0.12 | 0.03 | 0.24 | 0.31 | 0.08 | -0.07 | -0.21 | 0.12 | 0.09 | **0.64** |
| CBCL_70 | see things that aren't there | 0.15 | -0.16 | 0.14 | 0.15 | **0.7** | 0.01 | -0.22 | 0 | -0.05 | 0.19 | **0.58** |
| CBCL_71 | self-conscious | 0.08 | **0.46** | 0 | 0.21 | 0.16 | -0.11 | 0.27 | -0.14 | 0.13 | 0.13 | **0.58** |
| CBCL_74 | shows off | **0.5** | 0 | **0.38** | -0.23 | -0.08 | **0.42** | 0.08 | **0.32** | -0.1 | 0.05 | **0.45** |
| CBCL_75 | too shy | -0.13 | 0.3 | -0.06 | **0.38** | 0.31 | -0.3 | 0.03 | -0.31 | 0.19 | 0.14 | **0.5** |
| CBCL_76 | sleeps less | 0.03 | 0.26 | 0.31 | 0.04 | 0.23 | -0.03 | -0.06 | 0.11 | 0.05 | 0.23 | **0.51** |
| CBCL_77 | sleeps a lot | 0.08 | 0.01 | 0.16 | 0.14 | 0.28 | -0.04 | -0.02 | -0.02 | -0.01 | -0.03 | **0.41** |
| CBCL_79 | speech problem | -0.06 | -0.1 | **0.32** | 0.24 | 0.1 | -0.08 | -0.13 | 0.23 | 0.18 | -0.05 | 0.31 |
| CBCL_80 | stares blankly | -0.1 | -0.02 | **0.56** | 0.26 | 0.26 | -0.12 | -0.19 | 0.22 | 0.14 | 0.06 | **0.69** |
| CBCL_81 | steals at home | **0.66** | -0.27 | 0.07 | 0.11 | 0.25 | **0.52** | -0.2 | 0.03 | 0.03 | 0.11 | **0.52** |
| CBCL_82 | steals outside | **0.62** | **-0.33** | 0.11 | 0.12 | 0.31 | **0.44** | -0.26 | 0.06 | 0.01 | 0.01 | **0.55** |
| CBCL_83 | hoards things | 0.19 | 0.14 | 0.26 | 0.02 | 0.09 | 0.07 | 0.05 | 0.08 | 0.03 | 0.13 | **0.5** |
| CBCL_84 | strange behavior | 0.23 | 0.05 | **0.39** | 0.29 | 0.14 | 0.09 | -0.13 | 0.17 | 0.17 | 0.03 | **0.74** |
| CBCL_85 | strange ideas | 0.12 | 0.01 | **0.42** | 0.23 | 0.15 | 0.05 | -0.06 | 0.22 | 0.11 | 0.03 | **0.64** |
| CBCL_86 | stubborn, irritable | **0.58** | **0.35** | -0.05 | 0.04 | 0.11 | **0.39** | 0.14 | -0.16 | -0.03 | 0.08 | **0.65** |
| CBCL_87 | mood changes | **0.43** | **0.49** | 0.01 | 0.05 | 0.2 | 0.18 | 0.11 | -0.16 | -0.18 | -0.09 | **0.81** |
| CBCL_88 | sulks a lot | **0.42** | **0.38** | -0.04 | 0.16 | 0.08 | 0.19 | 0.21 | -0.19 | 0.02 | 0 | **0.68** |
| CBCL_89 | suspicious | **0.41** | 0.15 | 0.15 | 0.14 | 0.14 | 0.23 | 0.02 | -0.1 | -0.03 | -0.02 | **0.72** |
| CBCL_90 | swears | **0.6** | 0.02 | 0 | 0.08 | 0.07 | **0.42** | -0.08 | 0 | 0 | -0.01 | **0.54** |
| CBCL_91 | suicidality | **0.36** | **0.39** | 0.01 | 0.17 | 0.02 | 0.18 | 0.3 | 0.02 | 0.07 | -0.02 | **0.57** |
| CBCL_92 | sleep talks or walks | 0.08 | 0.08 | 0.23 | -0.16 | 0.17 | 0.08 | 0.11 | 0.17 | -0.07 | 0.18 | 0.21 |
| CBCL_93 | talks too much | 0.24 | 0.08 | **0.58** | -0.2 | -0.09 | 0.14 | 0.12 | **0.44** | -0.13 | -0.07 | **0.48** |
| CBCL_94 | teases a lot | **0.65** | 0.04 | 0.09 | -0.07 | 0.01 | **0.45** | 0.05 | 0.07 | -0.03 | 0.01 | **0.53** |
| CBCL_95 | temper tantrums | **0.65** | 0.32 | -0.04 | 0.04 | 0 | **0.46** | 0.12 | -0.12 | -0.11 | -0.02 | **0.65** |
| CBCL_97 | threatens people | **0.78** | 0.07 | -0.09 | 0.13 | 0.06 | **0.6** | 0.01 | -0.09 | 0.09 | 0.01 | **0.63** |
| CBCL_100 | trouble sleeping | 0.01 | **0.35** | 0.29 | -0.02 | 0.24 | -0.02 | 0.03 | 0.08 | 0.01 | 0.32 | **0.58** |
| CBCL_102 | slow moving | 0.08 | 0.03 | 0.11 | **0.4** | 0.22 | -0.16 | -0.03 | -0.08 | 0.21 | -0.06 | **0.61** |
| CBCL_103 | sad | 0.26 | **0.47** | -0.09 | 0.32 | 0.11 | 0.08 | 0.27 | -0.15 | 0.1 | -0.03 | **0.71** |
| CBCL_104 | unusually loud | **0.34** | 0.14 | **0.49** | -0.09 | -0.1 | 0.27 | 0.09 | 0.29 | -0.12 | -0.03 | **0.63** |
| CBCL_106 | vandalism | **0.62** | -0.1 | 0.15 | 0.01 | 0.29 | **0.43** | -0.18 | 0.07 | 0.04 | 0.04 | **0.57** |
| CBCL_107 | wets self during day | 0.13 | 0.03 | 0.1 | 0.14 | 0.15 | 0.06 | -0.03 | 0.12 | 0.2 | 0.08 | **0.39** |
| CBCL_108 | wets the bed | 0.2 | -0.07 | 0.07 | 0.05 | 0.05 | 0.13 | -0.08 | 0.15 | 0.07 | 0.06 | 0.19 |
| CBCL_109 | whining | **0.42** | 0.32 | 0.07 | -0.02 | -0.03 | 0.2 | 0.18 | 0.04 | -0.08 | 0.05 | **0.57** |
| CBCL_111 | withdrawn | 0.09 | 0.18 | -0.05 | **0.64** | 0.26 | -0.15 | -0.04 | -0.18 | **0.43** | 0.01 | **0.7** |
| CBCL_112 | worries | -0.04 | **0.65** | 0.14 | 0.06 | 0.05 | -0.13 | **0.45** | -0.03 | 0.07 | 0.25 | **0.58** |
| DISTRACT | distracted | 0.07 | 0 | 0.8 | 0.05 | 0.04 | 0.1 | -0.21 | **0.48** | 0 | 0.04 | **0.71** |
| DESTROYS | destroys | **0.62** | 0.03 | 0.12 | 0.1 | 0.14 | **0.41** | -0.14 | 0.08 | -0.03 | -0.03 | **0.67** |
| MANIA_1 | restless | 0.12 | 0.06 | **0.55** | -0.03 | 0.11 | 0.06 | -0.12 | 0.24 | -0.19 | -0.07 | **0.65** |
| MANIA_2 | unusually happy | 0.1 | 0.07 | 0.32 | 0.03 | 0.26 | 0.03 | -0.08 | 0.13 | -0.11 | -0.11 | **0.57** |
| MANIA_3 | rapid mood changes | 0.31 | **0.41** | 0.07 | 0.03 | 0.15 | 0.15 | 0.11 | -0.06 | -0.16 | -0.13 | **0.71** |
| MANIA_4 | anxious, tense | 0.12 | 0.18 | **0.42** | -0.02 | 0.2 | 0.01 | -0.02 | 0.15 | -0.19 | -0.06 | **0.67** |
| MANIA_5 | urge to destroy | **0.34** | 0.15 | 0.2 | 0.13 | 0.16 | 0.23 | -0.05 | 0.03 | -0.18 | -0.1 | **0.7** |
| MANIA_6 | insomnia | 0.16 | 0.11 | **0.37** | -0.02 | 0.19 | 0.05 | -0.04 | 0.16 | -0.14 | 0.04 | **0.64** |
| MANIA_7 | moods either up or down | 0.3 | **0.35** | 0.21 | 0.05 | 0.11 | 0.16 | 0.08 | 0.03 | -0.09 | -0.12 | **0.72** |
| MANIA_8 | overflowing with energy | 0.29 | 0.26 | 0.19 | 0.11 | 0.18 | 0.07 | 0.07 | -0.02 | -0.11 | -0.15 | **0.73** |
| MANIA_9 | irritable | 0.32 | 0.26 | 0.19 | 0.06 | 0.14 | 0.17 | 0.04 | -0.01 | -0.12 | -0.1 | **0.7** |
| MANIA_10 | racing thoughts | -0.01 | 0.15 | **0.62** | 0.04 | 0.01 | 0.03 | 0.02 | **0.34** | -0.02 | -0.01 | **0.61** |
| SSRS_6 | rather be alone | -0.1 | 0.11 | -0.09 | **0.57** | 0.17 | -0.11 | -0.04 | -0.15 | **0.38** | 0.05 | **0.37** |
| SSRS_15R | understands expressions and tone | 0.07 | -0.12 | 0.18 | 0.16 | 0.03 | 0.05 | -0.13 | 0.1 | 0.05 | -0.12 | 0.27 |
| SSRS_16 | avoids eye contact | -0.01 | -0.04 | 0.23 | **0.38** | 0.16 | -0.07 | -0.19 | 0.03 | 0.16 | 0.01 | **0.52** |
| SSRS_18 | difficulty making friends | 0.09 | 0.05 | 0.12 | **0.65** | -0.18 | 0.02 | 0.15 | 0.15 | **0.5** | -0.08 | **0.46** |
| SSRS_24 | resistant to change | 0.09 | 0.21 | 0.28 | 0.25 | 0.02 | 0.04 | 0.06 | 0.1 | 0.14 | 0.04 | **0.59** |
| SSRS_29 | seen as weird | 0.03 | 0.02 | **0.35** | **0.54** | -0.18 | 0.02 | 0.12 | 0.3 | **0.46** | -0.05 | **0.5** |
| SSRS_35 | trouble with flow of conversation | -0.08 | -0.06 | **0.51** | 0.32 | 0.04 | -0.06 | -0.13 | 0.31 | 0.18 | -0.07 | **0.54** |
| SSRS_37 | can't relate to peers | 0.06 | 0.06 | 0.27 | **0.56** | -0.14 | 0.04 | 0.07 | 0.18 | **0.46** | -0.08 | **0.51** |
| SSRS_39 | narrow range of interests | 0 | 0.01 | 0.26 | **0.38** | 0.13 | -0.04 | -0.11 | 0.09 | 0.24 | -0.07 | **0.53** |
| SSRS_42 | overly sensitive to sounds, touch | -0.07 | 0.2 | 0.3 | 0.18 | 0.13 | -0.1 | 0.05 | 0.08 | 0.07 | 0.09 | **0.5** |
| SSRS_58 | concentrates too much | 0.02 | 0.03 | **0.47** | 0.16 | 0.11 | -0.02 | -0.09 | 0.23 | 0.05 | -0.08 | **0.56** |

#

#### Table S5. Parent-report full sample B-ESEM

|  |  | **Parent-report full sample four-factor B-ESEM** | | | | |
| --- | --- | --- | --- | --- | --- | --- |
| **Item** | **Description** | **EXT** | **INT** | **THO** | **NDD** | **G** |
| CBCL_01 | too young | -0.03 | -0.13 | -0.09 | 0.08 | **0.64** |
| CBCL_03 | argues | **0.51** | 0.14 | -0.1 | 0.07 | **0.6** |
| CBCL_04 | fails to finish | 0.08 | -0.08 | 0.04 | 0.2 | **0.69** |
| CBCL_05 | little they enjoy | 0.09 | 0.1 | 0.14 | -0.21 | **0.66** |
| CBCL_06 | bowel movements | 0.05 | -0.06 | 0 | -0.04 | **0.37** |
| CBCL_07 | bragging | **0.36** | 0.04 | -0.11 | 0.25 | **0.4** |
| CBCL_09 | obsessions | -0.04 | 0.13 | -0.04 | 0.2 | **0.72** |
| CBCL_10 | restless | 0.09 | -0.12 | 0.08 | **0.46** | **0.7** |
| CBCL_11 | clings to people | -0.02 | 0.21 | -0.2 | 0.11 | **0.59** |
| CBCL_12 | lonely | 0 | 0.21 | -0.05 | -0.03 | **0.65** |
| CBCL_13 | confused | -0.2 | -0.02 | 0.18 | 0.09 | **0.72** |
| CBCL_14 | cries a lot | 0.12 | 0.25 | 0.12 | 0 | **0.57** |
| CBCL_15 | cruel to animals | **0.33** | -0.13 | 0.05 | -0.06 | **0.57** |
| CBCL_16 | cruel/bullies | **0.55** | -0.05 | -0.04 | -0.07 | **0.55** |
| CBCL_17 | daydreams | -0.21 | -0.03 | 0.12 | 0.15 | **0.62** |
| CBCL_18 | harms self | 0.18 | 0.22 | -0.05 | -0.14 | **0.58** |
| CBCL_19 | demands attention | 0.3 | 0.17 | -0.15 | 0.27 | **0.64** |
| CBCL_22 | disobedient at home | **0.6** | -0.02 | 0.03 | 0.05 | **0.61** |
| CBCL_23 | disobedient at school | **0.43** | -0.29 | 0.03 | 0.12 | **0.62** |
| CBCL_24 | doesn't eat well | 0.04 | 0.11 | 0.04 | -0.01 | **0.45** |
| CBCL_25 | doesn't get along well | 0.22 | -0.15 | -0.23 | -0.19 | **0.74** |
| CBCL_26 | doesn't feel guilty | **0.43** | -0.13 | 0.09 | 0.01 | **0.6** |
| CBCL_27 | easily jealous | **0.33** | 0.21 | -0.14 | 0.04 | **0.58** |
| CBCL_28 | breaks rules | **0.57** | -0.15 | 0.07 | 0.09 | **0.64** |
| CBCL_29 | fears animals | -0.09 | 0.31 | -0.09 | 0.06 | **0.44** |
| CBCL_30 | fears going to school | -0.1 | 0.27 | -0.15 | -0.13 | **0.57** |
| CBCL_31 | fears they will do bad | -0.05 | **0.4** | -0.19 | 0.01 | **0.51** |
| CBCL_32 | has to be perfect | -0.09 | **0.53** | -0.23 | -0.05 | **0.33** |
| CBCL_33 | feels no one loves them | 0.24 | 0.3 | -0.25 | -0.12 | **0.62** |
| CBCL_34 | others are out to get them | 0.19 | 0.2 | -0.18 | -0.1 | **0.67** |
| CBCL_35 | feels worthless | 0.03 | **0.34** | -0.27 | -0.11 | **0.64** |
| CBCL_36 | accident prone | -0.01 | 0.04 | 0 | 0.23 | **0.45** |
| CBCL_37 | gets into fights | **0.47** | -0.15 | -0.04 | -0.05 | **0.6** |
| CBCL_38 | gets teased | -0.02 | -0.08 | 0.11 | -0.09 | **0.67** |
| CBCL_39 | hangs out with trouble | **0.33** | -0.14 | 0.05 | 0.11 | **0.48** |
| CBCL_40 | hears sounds or voices | 0.05 | 0.02 | **0.41** | -0.03 | **0.55** |
| CBCL_41 | impulsive | 0.26 | -0.09 | -0.04 | 0.25 | **0.73** |
| CBCL_42 | rather be alone | -0.18 | 0.02 | 0.03 | **-0.45** | **0.61** |
| CBCL_43 | lying or cheating | **0.45** | -0.14 | 0.08 | 0.09 | **0.56** |
| CBCL_44 | bites fingernails | 0.02 | 0.06 | 0.02 | 0.09 | 0.3 |
| CBCL_45 | nervous | -0.06 | **0.41** | 0.12 | 0.12 | **0.66** |
| CBCL_46 | nervous movements | -0.09 | 0.12 | 0.1 | 0.23 | **0.6** |
| CBCL_47 | nightmares | 0.03 | 0.24 | 0.08 | 0.14 | **0.46** |
| CBCL_48 | not liked | 0.04 | -0.14 | **-0.32** | -0.17 | **0.77** |
| CBCL_49 | constipated | -0.04 | 0.2 | -0.03 | 0 | **0.34** |
| CBCL_50 | too fearful or anxious | -0.18 | **0.52** | 0.21 | 0.06 | **0.6** |
| CBCL_51 | feels dizzy | -0.05 | **0.33** | -0.18 | 0.04 | **0.4** |
| CBCL_52 | feels too guilty | -0.11 | **0.49** | **-0.38** | 0.04 | **0.52** |
| CBCL_53 | overeating | 0.05 | -0.05 | 0.05 | 0 | **0.38** |
| CBCL_54 | overtired without reason | -0.01 | 0.15 | **-0.42** | -0.08 | **0.56** |
| CBCL_55 | overweight | -0.02 | -0.11 | 0.14 | -0.07 | 0.2 |
| CBCL_56A | aches | 0.01 | 0.27 | 0.19 | 0.09 | 0.28 |
| CBCL_56B | headaches | 0.04 | 0.28 | 0.26 | 0.06 | 0.26 |
| CBCL_56C | nausea | 0.02 | **0.41** | 0.21 | 0.11 | 0.31 |
| CBCL_56D | problems with eyes | -0.05 | 0.12 | 0.09 | -0.02 | 0.3 |
| CBCL_56E | rashes or skin problems | 0.01 | 0.14 | 0.23 | 0.09 | 0.24 |
| CBCL_56F | stomachaches | 0.04 | **0.4** | 0.27 | 0.11 | 0.27 |
| CBCL_56G | vomiting | 0.05 | 0.17 | 0.18 | 0.11 | 0.22 |
| CBCL_56H | somatoform problems | 0 | 0.21 | -0.02 | 0.06 | **0.35** |
| CBCL_57 | attacks people | **0.54** | -0.02 | -0.02 | -0.15 | **0.57** |
| CBCL_58 | picks nose, skin | 0.05 | 0.03 | 0.2 | 0.11 | **0.45** |
| CBCL_60 | plays with sex parts too much | 0.15 | -0.08 | -0.02 | 0.11 | **0.4** |
| CBCL_61 | poor schoolwork | 0.09 | -0.24 | 0.05 | 0.12 | **0.66** |
| CBCL_62 | poor coordination | -0.15 | -0.08 | -0.02 | 0.17 | **0.63** |
| CBCL_63 | prefers older kids | 0.14 | 0.01 | 0 | 0.08 | **0.44** |
| CBCL_64 | prefers younger kids | -0.05 | -0.02 | -0.03 | 0.03 | **0.55** |
| CBCL_65 | refuses to talk | 0.05 | 0.11 | 0.18 | -0.3 | **0.59** |
| CBCL_66 | repeats acts | -0.02 | 0 | 0.09 | 0.11 | **0.71** |
| CBCL_67 | runs away | 0.31 | 0.06 | -0.04 | -0.05 | **0.65** |
| CBCL_68 | screams a lot | **0.43** | 0.13 | 0.12 | -0.01 | **0.61** |
| CBCL_69 | secretive | 0.12 | 0.11 | -0.03 | -0.2 | **0.58** |
| CBCL_70 | see things that aren't there | 0.05 | 0.03 | **0.48** | -0.05 | **0.55** |
| CBCL_71 | self-conscious | -0.06 | **0.39** | 0.17 | -0.15 | **0.53** |
| CBCL_72 | sets fires | 0.28 | -0.2 | -0.08 | 0.02 | **0.47** |
| CBCL_74 | shows off | **0.34** | -0.05 | 0.2 | **0.33** | **0.48** |
| CBCL_75 | too shy | -0.2 | 0.29 | 0.12 | **-0.33** | **0.43** |
| CBCL_76 | sleeps less | -0.03 | 0.14 | 0.15 | 0.1 | **0.54** |
| CBCL_77 | sleeps a lot | 0.01 | 0.05 | -0.13 | -0.04 | **0.41** |
| CBCL_79 | speech problem | -0.15 | -0.17 | 0.06 | 0.02 | **0.39** |
| CBCL_80 | stares blankly | -0.17 | -0.08 | 0.21 | 0.07 | **0.71** |
| CBCL_81 | steals at home | **0.47** | -0.23 | 0.24 | -0.03 | **0.56** |
| CBCL_82 | steals outside | **0.42** | -0.27 | 0 | -0.02 | **0.57** |
| CBCL_83 | hoards things | 0.07 | 0.1 | 0.04 | 0.09 | **0.49** |
| CBCL_84 | strange behaviour | 0.03 | -0.06 | 0.09 | 0 | **0.79** |
| CBCL_85 | strange ideas | -0.02 | -0.05 | 0.06 | 0.07 | **0.68** |
| CBCL_86 | stubborn, irritable | **0.42** | 0.25 | 0.06 | -0.07 | **0.62** |
| CBCL_87 | mood changes | **0.33** | **0.34** | -0.06 | -0.06 | **0.7** |
| CBCL_88 | sulks a lot | 0.25 | 0.3 | 0.03 | -0.13 | **0.62** |
| CBCL_89 | suspicious | 0.26 | 0.12 | 0.05 | -0.05 | **0.67** |
| CBCL_90 | swears | **0.41** | -0.05 | -0.05 | -0.03 | **0.53** |
| CBCL_91 | suicidality | 0.18 | 0.25 | 0.15 | -0.07 | **0.59** |
| CBCL_92 | sleep talks or walks | 0.04 | 0.11 | 0.06 | 0.21 | 0.24 |
| CBCL_93 | talks too much | 0.1 | -0.01 | -0.13 | **0.42** | **0.52** |
| CBCL_94 | teases a lot | **0.44** | -0.01 | -0.05 | 0.09 | **0.53** |
| CBCL_95 | temper tantrums | **0.49** | 0.19 | 0.01 | -0.03 | **0.62** |
| CBCL_96 | thinks about sex | 0.29 | -0.02 | -0.02 | 0.09 | **0.53** |
| CBCL_97 | threatens people | **0.56** | -0.02 | -0.1 | -0.12 | **0.64** |
| CBCL_98 | thumb-sucking | 0.06 | 0.02 | 0.04 | 0.04 | 0.14 |
| CBCL_100 | trouble sleeping | -0.03 | 0.26 | 0.03 | 0.12 | **0.55** |
| CBCL_102 | slow moving | -0.13 | 0.05 | 0.05 | -0.2 | **0.58** |
| CBCL_103 | sad | 0.11 | **0.34** | -0.09 | -0.21 | **0.66** |
| CBCL_104 | unusually loud | 0.21 | 0.03 | 0.18 | 0.31 | **0.64** |
| CBCL_106 | vandalism | **0.43** | -0.16 | -0.07 | 0.02 | **0.62** |
| CBCL_107 | wets self during day | -0.01 | -0.02 | 0.07 | -0.04 | **0.41** |
| CBCL_108 | wets the bed | 0.08 | -0.1 | 0.05 | 0.03 | 0.24 |
| CBCL_109 | whines | 0.25 | 0.23 | 0.09 | 0.07 | **0.52** |
| CBCL_111 | withdrawn | -0.13 | 0.08 | 0.08 | **-0.43** | **0.71** |
| CBCL_112 | worries | -0.12 | **0.56** | -0.1 | 0.02 | **0.53** |
| DESTROYS | destroys things | **0.41** | -0.07 | 0.04 | 0.02 | **0.69** |
| DISTRACT | distracted | -0.02 | -0.17 | 0.1 | **0.35** | **0.78** |
| MANIA_1 | restless | 0.08 | -0.03 | 0.12 | 0.26 | **0.62** |
| MANIA_10 | unusually happy | -0.05 | 0 | -0.01 | 0.27 | **0.64** |
| MANIA_2 | rapid mood changes | 0.06 | 0.03 | 0.12 | 0.11 | **0.54** |
| MANIA_3 | anxious, tense | 0.25 | 0.27 | 0.03 | -0.01 | **0.62** |
| MANIA_4 | urge to destroy | 0.07 | 0.11 | 0.11 | 0.18 | **0.62** |
| MANIA_5 | insomnia | 0.26 | 0.08 | 0.08 | 0.04 | **0.66** |
| MANIA_6 | moods either up or down | 0.09 | 0.08 | 0.11 | 0.17 | **0.58** |
| MANIA_7 | overflowing with energy | 0.21 | 0.19 | 0 | 0.04 | **0.67** |
| MANIA_8 | irritable | 0.16 | 0.18 | 0.03 | -0.01 | **0.67** |
| MANIA_9 | racing thoughts | 0.23 | 0.16 | 0.04 | 0.03 | **0.64** |
| SSRS_15R | understands expressions and tone | 0.01 | -0.15 | 0.04 | -0.01 | 0.28 |
| SSRS_16 | avoids eye contact | -0.09 | -0.09 | 0.15 | -0.13 | **0.53** |
| SSRS_18 | difficulty making friends | -0.14 | -0.1 | -0.25 | -0.25 | **0.58** |
| SSRS_24 | resistant to change | -0.01 | 0.07 | 0 | -0.01 | **0.61** |
| SSRS_29 | seen as weird | -0.18 | -0.14 | -0.23 | -0.08 | **0.64** |
| SSRS_35 | trouble with flow of convo | -0.17 | -0.17 | 0.05 | 0.07 | **0.61** |
| SSRS_37 | can't relate to peers | -0.13 | -0.12 | -0.19 | -0.16 | **0.64** |
| SSRS_39 | narrow range of interests | -0.1 | -0.09 | 0.07 | -0.12 | **0.57** |
| SSRS_42 | overly sensitive to sounds, touch | -0.11 | 0.12 | 0.07 | 0.02 | **0.5** |
| SSRS_58 | concentrates too much, repetitive | -0.06 | -0.07 | 0.07 | 0.11 | **0.6** |
| SSRS_6 | rather be alone | -0.17 | 0.03 | 0.07 | **-0.38** | **0.4** |

#### Table S6. Fit indices for all models.

|  | | **RMSEA** | **CFI** | **TLI** |
| --- | --- | --- | --- | --- |
| **Child-report** | **First Half ESEM** | | | |
|  | Two-factor | 0.028 | 0.889 | 0.882 |
|  | Three-factor | 0.024 | 0.920 | 0.914 |
|  | Four-factor | 0.021 | 0.941 | 0.935 |
|  | Five-factor | 0.018 | 0.955 | 0.948 |
|  | **Second Half B-ESEM** | | | |
|  | Four-factor | 0.018 | 0.956 | 0.950 |
|  | Five-factor | 0.016 | 0.964 | 0.959 |
|  | **Full Sample B-ESEM** | | | |
|  | Four-factor | 0.019 | 0.953 | 0.947 |
| **Parent-report** | **First Half ESEM** | | | |
|  | Two-factor | 0.020 | 0.931 | 0.927 |
|  | Three-factor | 0.018 | 0.947 | 0.943 |
|  | Four-factor | 0.016 | 0.958 | 0.955 |
|  | Five-factor | 0.015 | 0.963 | 0.960 |
|  | **Second Half B-ESEM** | | | |
|  | Four-factor | 0.015 | 0.966 | 0.962 |
|  | Five-factor | 0.014 | 0.963 | 0.960 |
|  | **Full Sample B-ESEM** | | | |
|  | Four-factor | 0.016 | 0.961 | 0.957 |

**Note:** RMSEA= root-mean-square error of approximation; CFI= comparative fit index; TLI=Tucker-Lewis index.

## References

1. [Achenbach, T. M. (1991). Manual for The Child Behavior Checklist/4-18 and 1991 Profile. *University of Vermont, Department of Psychiatry*.](http://paperpile.com/b/QBFBNP/XON6p) <https://ci.nii.ac.jp/naid/20001666977/>
2. [Achenbach, T. M., Mc Conaughy, S. H., Ivanova, M. Y., & Rescorla, L. A. (2011). *Manual for the ASEBA Brief Problem Monitor^TM^ (BPM)*.](http://paperpile.com/b/QBFBNP/kJUCp)
3. [Afzali, M. H., Sunderland, M., Carragher, N., & Conrod, P. (2018). The Structure of Psychopathology in Early Adolescence: Study of a Canadian Sample: La structure de la psychopathologie au début de l’adolescence: étude d’un échantillon canadien. *Canadian Journal of Psychiatry. Revue Canadienne de Psychiatrie*, *63*(4), 223–230.](http://paperpile.com/b/QBFBNP/hvekj)
4. [Bloemen, A. J. P., Oldehinkel, A. J., Laceulle, O. M., Ormel, J., Rommelse, N. N. J., & Hartman, C. A. (2018). The association between executive functioning and psychopathology: general or specific? *Psychological Medicine*, *48*(11), 1787–1794.](http://paperpile.com/b/QBFBNP/ilbi9)
5. [Caspi, A., Houts, R. M., Belsky, D. W., Goldman-Mellor, S. J., Harrington, H., Israel, S., Meier, M. H., Ramrakha, S., Shalev, I., Poulton, R., & Moffitt, T. E. (2014). The p Factor: One General Psychopathology Factor in the Structure of Psychiatric Disorders? *Clinical Psychological Science*, *2*(2), 119–137.](http://paperpile.com/b/QBFBNP/hYnJs)
6. [Colder, C. R., & O’Connor, R. M. (2004). Gray’s reinforcement sensitivity model and child psychopathology: laboratory and questionnaire assessment of the BAS and BIS. *Journal of Abnormal Child Psychology*, *32*(4), 435–451.](http://paperpile.com/b/QBFBNP/XC3nt)
7. [Cooper, A., Gomez, R., & Aucote, H. (2007). The Behavioural Inhibition System and Behavioural Approach System (BIS/BAS) Scales: Measurement and structural invariance across adults and adolescents. *Personality and Individual Differences*, *43*(2), 295–305.](http://paperpile.com/b/QBFBNP/7CC5C)
8. [Cowan, H. R., & Mittal, V. A. (2020). Transdiagnostic Dimensions of Psychiatric Comorbidity in Individuals at Clinical High Risk for Psychosis: A Preliminary Study Informed by HiTOP. *Frontiers in Psychiatry / Frontiers Research Foundation*, *11*, 614710.](http://paperpile.com/b/QBFBNP/UWBOm)
9. [Goldberg, L. R. (2006). Doing it all Bass-Ackwards: The development of hierarchical factor structures from the top down. *Journal of Research in Personality*, *40*(4), 347–358.](http://paperpile.com/b/QBFBNP/90cvb)
10. [Haltigan, J. D., Aitken, M., Skilling, T., Henderson, J., Hawke, L., Battaglia, M., Strauss, J., Szatmari, P., & Andrade, B. F. (2018). “P” and “DP:” Examining Symptom-Level Bifactor Models of Psychopathology and Dysregulation in Clinically Referred Children and Adolescents. *Journal of the American Academy of Child and Adolescent Psychiatry*, *57*(6), 384–396.](http://paperpile.com/b/QBFBNP/NLK5w)
11. [Katz, B. A., Matanky, K., Aviram, G., & Yovel, I. (2020). Reinforcement sensitivity, depression and anxiety: A meta-analysis and meta-analytic structural equation model. *Clinical Psychology Review*, *77*, 101842.](http://paperpile.com/b/QBFBNP/Jjt2L)
12. [Keyes, K. M., Eaton, N. R., Krueger, R. F., Skodol, A. E., Wall, M. M., Grant, B., Siever, L. J., & Hasin, D. S. (2013). Thought disorder in the meta-structure of psychopathology. *Psychological Medicine*, *43*(8), 1673–1683.](http://paperpile.com/b/QBFBNP/yKBvW)
13. [Kingsbury, A., Coplan, R. J., Weeks, M., & Rose-Krasnor, L. (2013). Covering all the BAS’s: A closer look at the links between BIS, BAS, and socio-emotional functioning in childhood. *Personality and Individual Differences*, *55*(5), 521–526.](http://paperpile.com/b/QBFBNP/BTXMB)
14. [Kotov, R., Ruggero, C. J., Krueger, R. F., Watson, D., Yuan, Q., & Zimmerman, M. (2011). New dimensions in the quantitative classification of mental illness. *Archives of General Psychiatry*, *68*(10), 1003–1011.](http://paperpile.com/b/QBFBNP/KWiCj)
15. [Laceulle, O. M., Vollebergh, W. A. M., & Ormel, J. (2015). The Structure of Psychopathology in Adolescence: Replication of a General Psychopathology Factor in the TRAILS Study. *Clinical Psychological Science*, *3*(6), 850–860.](http://paperpile.com/b/QBFBNP/ozWi2)
16. [Michelini, G., Barch, D. M., Tian, Y., Watson, D., Klein, D. N., & Kotov, R. (2019). Delineating and validating higher-order dimensions of psychopathology in the Adolescent Brain Cognitive Development (ABCD) study. *Translational Psychiatry*, *9*(1), 261.](http://paperpile.com/b/QBFBNP/oJwcq)
17. [Moore, T. M., Kaczkurkin, A. N., Leighton Durham, E., Jeong, H. J., McDowell, M. G., Dupont, R. M., Applegate, B., Tackett, J. L., Cardenas-Iniguez, C., Kardan, O., Akcelik, G. N., Stier, A. J., Rosenberg, M. D., Hedeker, D., Berman, M. G., & Lahey, B. B. (2020). Criterion Validity and Relationships between Alternative Hierarchical Dimensional Models of General and Specific Psychopathology. In *bioRxiv* (p. 2020.04.27.064303). https://doi.org/](http://paperpile.com/b/QBFBNP/wvrE8)[10.1101/2020.04.27.064303](http://dx.doi.org/10.1101/2020.04.27.064303)
18. [Noordhof, A., Krueger, R. F., Ormel, J., Oldehinkel, A. J., & Hartman, C. A. (2015). Integrating autism-related symptoms into the dimensional internalizing and externalizing model of psychopathology. The TRAILS Study. *Journal of Abnormal Child Psychology*, *43*(3), 577–587.](http://paperpile.com/b/QBFBNP/qlzEH)
19. [Paolillo, E. W., McKenna, B. S., Nowinski, C. J., Thomas, M. L., Malcarne, V. L., & Heaton, R. K. (2020). NIH Toolbox® Emotion Batteries for Children: Factor-Based Composites and Norms. *Assessment*, *27*(3), 607–620.](http://paperpile.com/b/QBFBNP/ub1Dw)
20. [Vaidyanathan, U., Patrick, C. J., & Iacono, W. G. (2012). Examining the overlap between bipolar disorder, nonaffective psychosis, and common mental disorders using latent class analysis. *Psychopathology*, *45*(6), 361–365.](http://paperpile.com/b/QBFBNP/hmXUT)
21. [Watts, A. L., Smith, G. T., Barch, D. M., & Sher, K. J. (2020). Factor structure, measurement and structural invariance, and external validity of an abbreviated youth version of the UPPS-P Impulsive Behavior Scale. *Psychological Assessment*, *32*(4), 336–347.](http://paperpile.com/b/QBFBNP/bWbSY)
22. [Zapolski, T. C. B., & Smith, G. T. (2013). Comparison of Parent versus Child-Report of Child Impulsivity Traits and Prediction of Outcome Variables. *Journal of Psychopathology and Behavioral Assessment*, *35*(3), 301–313.](http://paperpile.com/b/QBFBNP/V8NLw)
23. [Zapolski, T. C. B., Stairs, A. M., Settles, R. F., Combs, J. L., & Smith, G. T. (2010). The measurement of dispositions to rash action in children. *Assessment*, *17*(1), 116–125.](http://paperpile.com/b/QBFBNP/Q1FDI)
